# Moving to business – changes in physical activity and sedentary behavior after multilevel intervention in small and medium-size workplaces

**DOI:** 10.1186/s12889-017-4229-4

**Published:** 2017-04-17

**Authors:** Minna Aittasalo, Matleena Livson, Sirpa Lusa, Ahti Romo, Henri Vähä-Ypyä, Kari Tokola, Harri Sievänen, Ari Mänttäri, Tommi Vasankari

**Affiliations:** 1 UKK Institute for Health Promotion Research, P.O. Box 30, FI-33501 Tampere, Finland; 2 Finnish Sports Confederation, Valo, FI-00093 Helsinki, Finland; 3 Finnish Institute of Occupational Health, P.O. Box 486, FI-33101 Tampere, Finland; 4 Lahti Regional Sports Federation, Urheilukeskus, FI-15110 Lahti, Finland

**Keywords:** Physical activity, Sedentary behavior, Accelerometer, Workplace, Promotion, Multilevel, Intervention

## Abstract

**Background:**

Regular physical activity (PA) promotes and excessive sedentary behavior (SB) deteriorates health. Yet the Finnish working-aged population spends most of the day sitting. A 1-year Moving To Business (MTB) -intervention supported small and medium-size workplaces to combat sedentariness. This paper reports the changes in employees’ PA and SB from before MTB (baseline) to 1 year after baseline (follow-up).

**Methods:**

Twelve workplaces with a total of 396 employees participated. Each workplace nominated a team to promote PA and reduce SB at organizational, working unit and employee level. The teams were mentored regionally through meetings, workshop and tools. Changes in PA and SB were assessed with a questionnaire and an accelerometer. Wald Confidence Interval (Cl) for a difference of proportions with matched pairs was used in the questionnaire data (%-points with 95% CI) and linear mixed model in the accelerometer data (minutes and % of wear-time with 95% CI).

**Results:**

The mean age of the respondents to the questionnaire (*N* = 296; 75%) was 42.6 (SD 10.9), 64% were women, 95% had some education after high school, 74% worked in the day shift, 71% did sedentary work and 51% were overweight. The mean number of actions implemented in the workplaces was 6.8 and the multilevel approach was fully applied in 6 workplaces. Based on the questionnaire the time spent in SB decreased from baseline to follow-up 16% (95% CI −29 to −3) in total and 22% (−41 to −3) at work. The accelerometer showed daily increases of 33.7 min (15.3 to 52.1) and 6.8% (3.1 to 10.4) in total PA, 30.9 min (15.3 to 46.5) and 6.1% (2.9 to 9.2) in light PA and 673 (209 to 1139) more steps at work. Daily SB at work decreased 44.9 min (−68.0 to −21.8) and 7.6% (−11.9 to −3.2). Daily leisure PA declined 11.0 min (−24.9 to 2.9) and 3.2% (−6.2 to −0.2). Number of levels or actions had no effect on changes.

**Conclusions:**

Employees’ PA increased and SB reduced at work during the intervention. At the same time leisure PA decreased slightly. Workplaces can achieve meaningful changes in employees’ PA and SB if assisted systematically. Controlled studies are needed to confirm the present findings.

**Trial registration:**

NCT01999205, registration date 11/01/2013.

**Electronic supplementary material:**

The online version of this article (doi:10.1186/s12889-017-4229-4) contains supplementary material, which is available to authorized users.

## Background

To preserve cardio-vascular health, every working-aged adult should be engaged in moderate-intensity physical activity (PA) for at least 150 min a week [1]. However, only 25 to 30% of the Finnish working-aged population meets this endurance part of the PA recommendation [2].

Being less active than recommended (i.e. being inactive) increases the risk for several chronic diseases such as type 2 diabetes, coronary heart disease, stroke, and colon cancer [3]. Recently it has been discovered that beyond inactivity, excessive prolonged sitting or more broadly sedentary behavior (SB) defined as “any waking behavior characterized by an energy expenditure ≤1.5 METs while in a sitting or reclining posture“[4] deteriorates health irrespective of meeting the PA recommendation [5–8]. Yet the Finnish working-aged population is sedentary almost two thirds of their day [2], which is the situation also in many other developed countries [9, 10].

Workplaces have been identified as an important forum for health promotion because they offer a setting for reaching large groups of working-aged population and enable utilization of existing social networks [11]. However, the impacts of workplace health promotion on, for example, employees’ weight, dietary habits, work absences and productivity have generally been small, short-lived or inconclusive [12], which applies also to PA promotion interventions [13–15]. The findings on reducing SB seem more promising and consistent especially when it comes to the use of sit-stand desks and active workstations [16, 17]. Results from interventions using larger diversity of strategies to reduce SB are not quite as optimistic [18–20].

To promote worksite health more productively, documents outlining best practices [21, 22], scientific reviews [23], expert opinions [24–26] and individual studies [27] all advocate the use of socio-ecological models [28, 29]. These models suggest that people’s health behavior is influenced not only by their intrapersonal characteristics but also by a wide variety of factors in their social, environmental and policy contexts. Thus, to achieve change in health behavior, actions should be targeted simultaneously at multiple levels. A multilevel approach is also thought to benefit in reaching such groups of people, who may remain underrepresented in single-level interventions [29]. For instance, in worksite PA promotion, people may be reached more effectively through modifying their physical environment at work to favor physical activity rather than providing them with personal incentive for leisure exercise.

The use of a multilevel approach seems particularly justified to promote PA and reduce SB since both behaviors are strongly connected with specific contexts such as schools, workplaces and homes [30]. Moreover, several previous studies indicate that the determinants of PA [31–35] and SB [36–39] are multilevel in nature. Also, studies examining the effectiveness of interventions on PA promotion [30, 40, 41] and SB reduction [19, 42, 43] speak for the use of multilevel approach.

However, so far the application of multilevel approach to health promotion and PA interventions has been limited [23, 44–46]. It seems that the empirical evidence on the effectiveness of multilevel interventions in relation to single-level interventions is yet unclear [44], which may be one reason for the limited use. As to the worksite setting in particular, only one review [47] has compared the effectiveness of single and multilevel strategies in changing employees’ PA behavior. The review showed most promise for interventions, which combined both environmental, policy and individual-level strategies.

The aim of this 1-year Moving To Business (MTB) intervention was to 1) support small and medium-size workplaces to plan and implement a multilevel intervention to increase PA and reduce SB among employees and to 2) evaluate the implementation and outcomes of the intervention. This paper reports the planning and implementation of the intervention as well the changes in PA and SB from baseline to 1-year follow-up.

## Methods

MTB was designed and evaluated by two research organizations, The UKK Institute for Health Promotion Research and the Finnish Institute of Occupational Health, and was implemented by the Finnish Sports Confederation in collaboration with three regional sports federations. The study plan was approved by the Ethics Committee of the Tampere Region, under the auspices of University of Tampere, Human Sciences (http://www.uta.fi/english/research/ethics/review/committee.html, running number 17/2013).

### Workplaces and employees

Regional contact persons from the collaborating regional sports federations recruited three to five small or medium-size workplaces to MTB. According to Finnish criteria enterprises with fewer than 50 employees are defined small and those with 50–249 employees medium-size [48]. During the recruitment process the contact persons kept record of the workplaces approached, recruitment methods used and the reasons given by the workplaces for not participating in MTB.

The workplaces accepting the invitation paid a participation fee of 2000 or 3000 Euros depending on their size. The fee engaged the workplaces in MTB and covered some of the implementation costs. For the additional commitment the workplaces signed a written contract with the Finnish Sports Confederation. The workplaces were also urged to contact their occupational health care provider to facilitate their participation in MTB. However, the workplaces were not obliged to do so because especially in small workplaces it may have required expansion of their existing occupational health care contracts leading to extra costs.

### Intervention

The MTB intervention comprised starting, active and closing phases. For the execution of the intervention each workplace nominated an internal MTB team involving members from the management, human resources, working staff and desirably also from occupational health care. With the support of regional contact persons the MTB teams specified the goals for increasing PA and reducing SB among employees and planned and implemented actions at organization, working unit and individual employee level to reach the goals.

*At the starting phase* (January 2014), a 4-hour opening meeting was arranged for the MTB teams by their regional contact person to discuss the needs and current practices for promoting PA and reducing SB at each particular workplace (Table 1). Results from the baseline measurements (November 2013) were presented to help the teams in goal setting and action planning. Also, the teams were provided with a planning sheet, which they used for scheduling the multilevel actions.

**Table 1:**
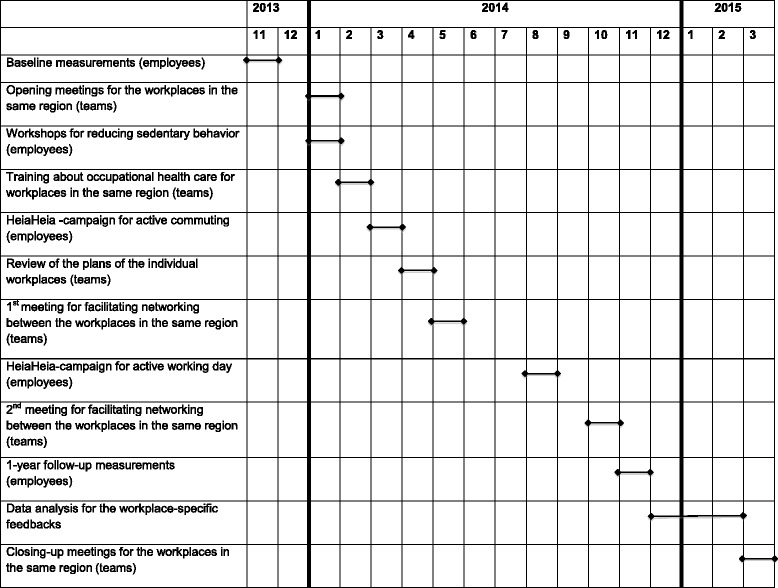
Pre-scheduled actions and measurements by month during 2013–15 in the Moving To Business (MTB) –intervention

*At the active phase* (February – October 2014) the regional contact persons provided the MTB teams with support and tools to implement the actions: a 4-hour training on occupational health care’s role in workplace health promotion, two meetings for the MTB teams in the same area to facilitate their networking and one workplace-specific meeting to preview the multilevel action plans. In addition, the MTB teams had an opportunity to get extra support from the regional contacts upon request (face-to-face and telephone consultation, group exercise services, help and material for organizing campaigns etc.). Moreover, the employees of the participating workplaces were offered a one to two-hour workshop on reducing SB and a possibility to use free-of-charge an internet-based platform (https://www.heiaheia.com) to monitor their PA and to share the information with their workmates and friends. Two separate Heiaheia-campaigns were also provided for the MTB teams to be delivered in the workplaces (“Active commuting to work” and “Active working day”).

*At the closing phase* (March 2015), a 4-hour closing-up meeting was arranged for the MTB teams located in the same regional area to share general and workplace-specific feedback on implementation and results.

The regional contact persons reported monthly on the progress in their workplaces with a structured form to the MTB coordinators (AR, ML) at the Finnish Sports Confederation. The coordinators, in turn, updated the developments to the MTB core group, which had also representatives from the research organizations (MA, SL).

### Measures

MTB targeted at all the employees in the participating workplaces. However, completing the baseline and follow-up measures was voluntary for ethical reasons. The follow-up measurements were repeated 1 year after the baseline the same time of the year (November) for seasonal comparability.

#### Questionnaire

At baseline the employees completed a self-administered questionnaire on their demographics, work, work ability, work engagement and recovery, PA, SB (sitting), perceived health, smoking and sleep. The questions on the quantity of PA and SB were the same as used in Finnish population surveys [49] (Additional file 1). The SB questions in the surveys are identical to the Workforce Sitting Questionnaire, which has been found valid for assessing sitting time at work as well as total sitting time during working and non-working day [50]. Compliance with the questionnaire was facilitated in the cover letter by emphasizing that the evaluation focused on the multilevel intervention, not on individual employees.

#### Accelerometer

PA and SB (sitting + reclining posture) were objectively assessed with a hip-worn accelerometer (Hookie AM13, Traxmeet Ltd., Espoo, Finland, http://company.traxmeet.com), which employed a triaxial sensor component (ADXL345; Analog Devices, Norwood MA). This device has been found as valid as the most commonly used accelerometer (Actigraph GTX3, Actigraph LLC, Pensacola FL, USA, http://actigraphcorp.com) in assessing adults’ PA and SB [51]. The employees were provided with written instructions to wear the accelerometer on their right hip during the waking hours for seven consecutive days and to remove the device only for sauna, shower and water activities.

To facilitate the accelerometer use, the employees were offered a graphical feedback about their PA and SB after the baseline and follow-up measurements. The MTB teams were also provided with graphics about PA and SB in the entire workplace. The baseline graphics were also utilized for goal setting and action planning.

#### Diary

A diary was used to extract specific data on working days and hours from the accelerometer data accumulated. On each accelerometer day the employees were asked to enter the following information to the day-specific row: date, working day/non-working day, time leaving home and arriving at work (hours: minutes), transportation modes used (1 = walking, 2 = cycling, 3 = other active mode, 4 = car, 5 = bus, 6 = train, 7 = moped or motorcycle, 8 = other motorized mode), time starting and ending the work (hours: minutes), time leaving work and arriving at home (hours: minutes), and transportation modes used.

### Data analysis

The acceleration data were collected in raw mode, which presents the acceleration data in actual G-force units (milligravity, mg). The accelerometer collected the data with a 100 Hz sampling frequency and a ± 16,000 mg dynamic range. After the measurement period the stored data were transferred to a hard disk and analysed in 6-s epochs.

Earlier studies indicate that 4 days with at least 10 h from each day is the minimal amount of data needed for reliable estimates of adults’ weekly PA with hip-worn monitors [52, 53]. Accordingly data from at least three working days with the minimum of 10 h wear-time was considered sufficient to describe employees’ PA and SB during regular working days. This amount of data covered 60% of the working days during a regular 5-day working week. In addition, 4 hours of wear-time was required during the working hours equaling 50% of the normal 8-h working day. Non-wear time was defined as no movement detected in any epoch for at least 30 min.

Accelerometer-specific cut-points, which have been previously determined from adults’ raw acceleration data by using mean amplitude deviation (MAD), were used to classify the intensity of employees’ intensity-specific PA (light, moderate, vigorous) and to separate SB (sitting + reclined posture) from PA [54]. Transition from sitting to standing posture was calculated on the basis of the number of SB periods ending with standing up. Standing was detected if the MAD value was greater than 50 mg for the preceding or same epoch when the measured posture changed to standing. [Vähä-Ypyä H, Husu P, Suni J, Vasankari T: Reliable recognition of lying, sitting and standing with a waist waist-worn accelerometer, submitted].

Descriptive information is presented as means and standard deviations (SD). Changes in the questionnaire-based PA and SB (sitting) variables from baseline to 1-year follow-up are reported in percentage points (%-points) and their 95% confidence intervals (95% CI) calculated with Wald Confidence Interval for a difference of proportions with matched pairs. The analysis included employees, who had questionnaire-based PA and SB data from both time points.

The accelerometer data at baseline and follow-up as well as the changes from baseline to follow-up are reported in mean minutes (or steps) and percentages of wear-time (% wear-time). Linear mixed model was used in analyzing the changes in employees, who had data from both time points. The outcome was the difference between the follow-up and baseline value of each specific variable. The baseline value was used as a confounding factor and the workplace served as a random effect. Sex, age (continuous), perceived health (poor or fairly poor; average; good or fairly good), physical exertion of the work (sedentary work; work with light or moderate mobility; heavy or extremely heavy physical work), working time (regular day shift; shift work without nightshifts; other) and education (basic education, high school or vocational school; university of applied sciences; university) were added as potential confounding factors in the model but removed one by one if they did not improve the model’s Bayesian Information Criterion [55] and were not statistically significant (*p* < 0.05). In addition, the change in wear-time was taken into account when analyzing the changes in mean minutes.

Linear Mixed Models with workplace as random effect were used to test whether the changes in PA and SB were different between the workplaces implementing more actions and fewer actions than on average and between the workplaces implementing actions at all three levels and at one or two levels.

Changes in PA and SB at individual workplaces were not analyzed because the number of employees having both baseline and follow-up accelerometer data was too small in the majority of workplaces for statistical or pragmatic conclusions (less than 10 employees in 5 workplaces, less than 20 in 5 workplaces, 38 in one workplace and 42 in one workplace).

## Results

### Participants

The three regional contact persons approached altogether 18 workplaces. Of them, 12 (67%) agreed to participate in the study (Table 2). One regional contact person succeeded in enrolling 3, one 4 and one 5 workplaces. The most common reasons for refusing participation were lack of time and resources as well as too many concurrent projects. The total number of employees in the participating workplaces was 396 ranging from 13 to 107 by individual workplace.

**Table 2:** Participating workplaces, their field of activity, number of employees, respondents to the questionnaire, employees with valid accelerometer data (Acc)^a^ and employees with valid accelerometer data and diary entries (Acc + D)^b^ at baseline and follow-up

		Baseline				Follow-up			
Workplace	Field of activity	Employees N (%)	Respondents N (%)	Acc^a^ N (%)	Acc + D^b^ N (%)	Employees N (%)	Respondents N (%)	Acc^a^ N (%)	Acc + D^b^ N (%)
1	Banking services	21 (5.3)	19 (90.5)	16 (76.2)	16 (76.2)	18 (5.0)	12 (66.7)	10 (55.6)	10 (55.6)
2	Climate control (HVAC)	30 (7.6)	22 (73.3)	18 (60.0)	18 (60.0)	28 (7.8)	19 (67.9)	16 (57.1)	16 (57.1)
3	Congress and concert center	23 (5.8)	20 (87.0)	18 (78.3)	18 (78.3)	21 (5.8)	17 (81.0)	16 (76.2)	16 (76.2)
4	Regional marketing	13 (3.3)	11 (84.6)	11 (84.6)	10 (76.9)	11 (3.1)	10 (90.9)	9 (81.8)	9 (81.8)
5	Media house	17 (4.3)	11 (64.7)	12 (70.6)	12 (70.6)	16 (4.5)	8 (50.0)	8 (50.0)	8 (50.0)
6	Banking services	107 (27.0)	79 (73.8)	73 (68.2)	73 (68.2)	98 (27.3)	52 (53.0)	44 (44.9)	44 (44.9)
7	Training institute	31 (7.8)	25 (80.6)	23 (74.2)	23 (74.2)	25 (7.0)	15 (60.0)	13 (52.0)	13 (52.0)
8	Technology center	20 (5.1)	11 (55.0)	10 (50.0)	10 (50.0)	16 (4.5)	8 (50.0)	6 (37.5)	6 (37.5)
9	Media house	19 (4.8)	14 (73.7)	12 (63.2)	12 (63.2)	15 (4.2)	9 (60.0)	7 (46.7)	7 (46.7)
10	Amusement park	30 (7.6)	27 (90.0)	27 (90.0)	27 (90.0)	29 (8.1)	14 (48.3)	12 (41.4)	12 (41.4)
11	Theater	16 (4.0)	11 (68.8)	8 (50.0)	8 (50.0)	16 (4.5)	10 (62.5)	8 (50.0)	8 (50.0)
12	Congress and concert center	69 (17.4)	46 (66.7)	38 (55.1)	37 (53.6)	66 (18.4)	31 (47.0)	26 (39.4)	26 (39.4)
Total		396 (100)	296 (74.7)	266 (67.2)	264 (66.7)	359 (100)	205 (57.1)	175 (48.7)	175 (48.7)

^a)^The minimum of 10 h of wear-time during at least 3 working days

^b)^The minimum of 10 h of wear-time during at least 3 working days and successful diary entries from the same days

The baseline questionnaire was responded by 295 (74%) employees and 266 (67%) had valid accelerometer and diary information. At 1-year follow-up all the workplaces enrolled were still involved but the number of employees in the workplaces had reduced by 37 (9% out of 396) leaving 359 persons to the follow-up sample. Of them, 201 (56%) responded to the follow-up questionnaire and 175 (49%) had relevant accelerometer and diary data.

The mean age of the respondents to the baseline questionnaire was 42.6 (SD 10.9), 64% were women, 95% had some education after high school, 74% worked in the day shift, 71% did sedentary work and 51% were overweight. More specific information on the baseline characteristics is given in Table 3.

**Table 3:** Baseline characteristics of the respondents to the baseline questionnaire (*n* = 296, 75%)

Age (years), mean (SD)	42.6 (10.9)
Age-group, n(%)	
< 25 years	9 (3.0)
25-44 years	154 (52.0)
45-54 years	89 (30.1)
≥ 55 years	44 (14.9)
Women, n (%)	189 (63.9)
Married, n (%)	222 (75.0)
Caretaker to children under 18 years of age, n (%)	135 (45.6)
Education, n (%)	
High school	16 (5.4)
Polytechnic or vocational school	198 (67.4)
University degree	77 (26.2)
Other	3 (1.0)
Working time, n (%)	
Regular day work	218 (73.9)
Shift-work (2 or 3 shifts)	52 (17.6)
Irregular or other hours	25 (8.5)
Type of work, n (%)	
Sedentary work	207 (70.7)
Mainly standing or light ambulatory work without carrying	23 (7.8)
Mainly ambulatory work with carrying or climbing stairs	46 (15.7)
Heavy or extremely heavy physical work	17 (5.8)
Body mass index (kg/m^2^), mean (SD)	25.6 (4.5)
Body mass index >25, n (%)	150 (50.7)
Cigarette smoking (yes), n (%)	46 (15.8)
Weekly portions of alcohol, mean (SD)	4.0 (5.5)

Reported as means and standard deviations (SD) or numbers (N) and proportions (%)

Both baseline and 1-year follow-up data were obtained with the questionnaire from 186 employees (47% from the original sample of 396 employees) and with the accelerometer and diary from 147 (37%) employees. The employees completing both the baseline and follow-up questionnaire were similar to those responding only to the baseline questionnaire in terms of sex, age, health, physical exertion of the work, working time, education, PA and SB (Additional file 2). Similarly, there was no statistical difference for sex, age, health, physical exertion of the work, working time, education, PA, SB between the employees, who completed the accelerometer and diary measurements only at baseline compared to those with data from both time points (Additional file 2).

### Implementation

All workplaces nominated the MTB teams, made actions plans for promoting PA and reducing SB and participated in the follow-up measurements. The size of the MTB teams ranged from 2 to 8 members. All 12 MTB teams included at least one representative from HR, working staff and occupational safety. Nine teams included also at least one member from the management, and 4 teams from the occupational health care.

Nearly all (11/12) workplaces aimed primarily at reducing SB. Sit-stand workstations, exercise equipment for collective use and opportunity to experience different modes of instructed exercise were among the most common actions implemented by the MTB teams (Table 4). The total number of various actions during MTB was 39: 16 (41%) focused on organization, 15 (38%) on working unit and 8 (21%) on individual employee. The mean number of actions implemented in the workplaces was 6.8 ranging from 2 to 11. Multilevel implementation at all three levels was applied in six workplaces (1, 4, 5, 7, 11, 12), while five workplaces (2, 3, 6, 9, 10) implemented actions at two levels and one workplace (8) at one level.

**Table 4:** Actions^a^ implemented at the three levels (organization, working unit, employee) in the participating workplaces for increasing physical activity and reducing sedentary behavior

Actions by level	Actions implemented in the 12 workplaces (1–12)											
	1	2	3	4	5	6	7	8	9	10	11	12
ORGANIZATION												
Providing staff with Sit and Stand desks			X	X	X		X			X	X	X
Standing desks in the meeting rooms and collective spaces									X		X	
Exercise equipment for the collective use			X	X	X		X		X		X	
Shared bicycle for work-related errands											X	
Nutritional campaign in collaboration with the restaurant staff										X		
Replacing of copy machines to increase daily steps												X
Stair posters beside the elevators						X						X
Documenting breaks from sitting in the organization strategy												X
Introducing walking routes for coffee breaks			X									
Providing stability balls for seats			X				X					
Updating the locker rooms			X									
Organizing physical activity day/days	X		X									
Engaging occupational health care in physical activity promotion	X		X		X	X						
Nominating a wellbeing team	X				X							
Incentive to use one hour working time per week for physical activity					X							
Purchasing saddle stools					X							
WORKING UNIT												
Arranging instructed physical activity breaks					X						X	X
Standing up during meetings (staff)							X		X		X	
Providing information on wellbeing in the folders placed in the coffee rooms	X										X	X
Having stand-up meetings (management group)												X
Offering lectures on physical activity, diet, rest etc.			X	X			X			X		X
Providing instruction sheets for physical activity breaks				X								
Giving guidance on how to use physical activity equipment				X								
Providing a possibility for physical activity experiments	X		X	X	X		X					
Arranging fitness tests/impedance measurements	X	X		X			X					
Having walking and stand-up meetings (staff)	X		X		X							
Reminding about and encouraging to stand in weekly meetings								X				
Having walking meetings							X	X				
Arranging stand-up coffee breaks during meetings								X				
Campaigning for active commuting to work								X				
Arranging weekly group-exercise							X					
INDIVIDUAL												
Wall pictures reminding about breaks from sitting				X							X	X
Breaks from sitting at one’s workstation during newscast					X							
Instructed exercise program for physical activity breaks						X	X					
E-mail messages and information on increasing physical activity												X
Including conversation on physical activity and rest in individual development discussions												X
Activity monitors to all employees	X											
Individual physical activity counseling based on impedance measurements	X	X										
Individual goals for bicycling							X					
Total number of actions per workplace	9	2	10	8	10	3	11	4	3	3	8	11

^a^Information collected from the action-plans of the workplaces. Minor revisions made by the authors for better correspondence of actions and levels

### PA and SB

#### Questionnaire

Table 5 presents the questionnaire-based findings on employees’ weekly PA and SB (sitting) at baseline, 1-year follow-up and changes from baseline to 1-year follow-up. At baseline the employees reported an average of 240 min of weekly leisure PA. The mean total duration of daily SB was 509 min, which was mostly (305 min) accumulated at work. At follow-up, the daily self-reported SB at work reduced from baseline to 275 min denoting a 22% (95% CI −41% to −3%) decrease. At the same time the proportion of active commuters decreased by 6% (95% CI −11% to −2%). No significant changes were observed in the weekly leisure PA or in daily breaks in sitting at work. Neither were there any changes in the amount of SB during transportation or leisure (TV, video, computer, other).

**Table 5:** Questionnaire-based employees’ physical activity (PA), sitting and daily breaks from sitting at baseline and at 1-year follow-up

Variables	Baseline	1-year follow-up	Change	
	*N* = 296	*N* = 205	*N* = 186	
			%-units	95% CI
Weekly leisure PA, mean (SD)				
• Total minutes	240 (222)	243 (248)	16	−67 to 98
• Light minutes	87 (145)	107 (195)	18	−51 to 87
• Moderate minutes	98 (145)	95 (132)	−8	−45 to 30
• Vigorous minutes	50 (81)	37 (58)	−4	−24 to16
• Muscle or balance training minutes	69 (105)	66 (87)	−21	−50 to 9
Daily sitting, mean (SD)				
• Total minutes	509 (142)	461 (159)	−16	−29 to −3
• Minutes at work	305 (140)	275 (146)	−22	−41 to −3
• Minutes in transportation	41 (41)	38 (40)	−13	−44 to 18
• Minutes during leisure^b^	163 (89)	149 (79)	−5	−26 to 16
Daily breaks in sitting at work, N (%)				
• Possibility to break sitting	218 (75)	149 (75)	1	−5 to 6
• Break once in half an hour	54 (19)	38 (19)	3	−4 to 10
• Break once in an hour	114 (39)	85 (43)	2	−7 to 11
Active work-commuting, N (%)				
• Walking or cycling	98 (33)	62 (30)	−6	−11 to −2

Change from baseline to follow-up in percentage points (%-points) and their 95% confidence intervals (95% CI)^a^

^a^Wald Confidence Interval for a difference of proportions with matched pairs

^b^TV, video, computer, other

#### Accelerometer

Table 6 presents the accelerometer-based findings on daily PA and SB (sitting + reclined posture) at baseline, 1-year follow-up and changes from baseline to 1-year follow-up.

**Table 6:** Accelerometer-based employees’ physical activity (PA) and sitting during a working day (working time + leisure time) at baseline and 1-year follow-up in mean minutes ($\overset{-}{x}$) and their standard deviations (SD) and change from baseline to 1-year follow-up^a^ in mean minutes ($\overset{-}{x}$)^b^ and percentages of wear-time (% wear-time) and their 95% confidence intervals (95% CI)

PA and sitting	Baseline (*N* = 266)		1-year follow-up (*N* = 175)		Change in mean minutes		Change in % wear-time	
	$\overset{-}{x}$ (SD)	% wear-time (SD)	$\overset{-}{x}$ (SD)	% wear-time (SD)	$\overset{-}{x}$	95% CI	% wear-time	95% CI
Working time								
PA								
• Total minutes	110.7 (74.4)	22.5 (14.3)	115.5 (73.6)	23.9 (14.2)	33.7	15.3 to 52.1	6.8	3.1 to 10.4
• Number of steps	3802 (2377)	-	3968 (2354)	-	673	209 to 1139	-	-
• Light minutes	89.7 (62.2)	18.2 (12.2)	92.3 (59.3)	19.2 (11.6)	30.9	15.3 to 46.5	6.1	2.9 to 9.2
• Moderate-to-vigorous minutes	21.0 (17.8)	4.2 (3.4)	23.2 (20.9)	4.8 (4.0)	4.1	−1.5 to 9.6	0.9	−0.2 to 2.0
Sitting (+ lying in reclined posture)								
• Total minutes	298.5 (81.3)	61.9 (16.6)	271.3 (79.2)	57.5 (16.7)	−44.9	−68.0 to −21.8	−7.6	−11.9 to −3.2
Breaks from sitting								
• Number of breaks	24.1 (9.5)	-	22.5 (8.8)	-	−0.2	−3.0 to 3.4	-	-
Leisure time								
PA								
• Total minutes	126.6 (41.7)	30.9 (9.0)	120.5 (36.4)	28.8 (7.9)	−11.0	−24.9 to 2.9	−3.2	−6.2 to −0.2
• Number of steps	4952 (2830)	-	4817 (2642)	-	−534	−1565 to 496		
• Light minutes	97.0 (32.8)	23.6 (6.9)	92.0 (29.4)	21.9 (5.9)	−8.1	−19.3 to 3.1	−2.4	−4.8 to 0.0
• Moderate-to-vigorous minutes	29.6 (20.2)	7.2 (5.0)	28.5 (18.3)	6.9 (4.7)	−2.7	−9.4 to 4.0	−0.5	−1.9 to 1.0
Sitting (+ lying in reclined posture)	246.9 (75.7)	59.3 (10.6)	255.9 (74.9)	60.1 (9.3)	12.4	−7.2 to 32.0	1.5	−2.1 to 5.1
Total PA (working + leisure time)	237.3 (80.7)	26.4 (8.8)	236.0 (76.1)	26.4 (8.7)	24.0	4.6 to 43.3	2.4	1.2 to 3.7

^a^Linear mixed model including employees, who had data from both time points

^b^Baseline value as a confounding factor, workplace as a random effect, adjustment for the change in wear-time from baseline to follow-up. Sex, age, perceived health, physical exertion of the work, working time and education added as confounding factors but removed one by one if they did not improve the model’s Bayesian Information Criterion and were not statistically significant (*p* < 0.05)

At baseline the employees were physically active at work on average 111 min a day, took 3802 steps, spent 299 min in SB and had 24 breaks from SB. During leisure, they had on average 127 min daily PA with 4952 steps and they spent 245 min in SB. At follow-up, the employees’ daily PA at work increased on average 34 min (95%CI 15.3 to 52.1) and 6.8% (95%CI 3.1 to 10.4) of wear-time compared with baseline. The employees also increased their daily steps by 673 (95%CI 209 to 1139) from baseline. In addition, their daily light-intensity PA increased 31 min (95%CI 15.3 to 46.5) and 6.1% (95%CI 2.9 to 9.2) of wear-time. Moreover, daily SB at work decreased 45 min (95% CI −68.0 to −21.8) and 7.6% (95%CI −11.9 to −3.2) of wear-time. However, at the same time, the total minutes of daily leisure PA decreased 11 min (95%CI −24.9 to 2.9) and 3.2% (95%CI −6.2 to −0.2) of wear-time.

#### Changes in PA and SB in relation to implementation

No significant differences were observed in changes in PA or SB between the workplaces implementing more or fewer actions than on average (6.8) or between the workplaces implementing actions at all three levels and just one or two levels (Additional file 3).

## Discussion

The purpose of the MTB intervention was to support small and medium-size workplaces to plan and implement multilevel intervention to increase PA and reduce SB among employees and to evaluate the implementation and outcomes of the intervention.

### Summary of key findings

During MTB, the employees’ self-reported SB (sitting) at work and in total decreased. Also objectively measured SB at work decreased and the minutes spent in total and light-intensity PA at work increased, which was also seen as the higher number of steps taken at work. If sustained, the magnitudes of these changes may be relevant for health although light-intensity PA contributed most of the increase in PA at work and was presumably also the primary replacement for SB. Nevertheless, recent studies indicate that light activity is better than SB for health [56].

Employees seemed to compensate part of their increased PA at work with a decrease in leisure PA, since the objectively measured total minutes of leisure PA were somewhat lower at follow-up compared with baseline. However, the mean decrease in leisure PA (−11 min) did not reach statistical significance and was clearly less than the concurrent increase in PA at work (+33.7 min). Yet this finding supports previous studies indicating a similar compensatory effect [57] and may pertain to the risk compensation theory, which suggests that a positive change achieved at one level (at work) can be replaced with opposite behaviour at another level (leisure) if the change is not facilitated simultaneously at both levels [44]. This may also explain the decrease in the self-reported proportion of active commuters at least to some extent, since only one workplace (8) with 20 employees implemented actions to promote active commuting to work apart from the HeaiHeia –campaign arranged in all the workplaces.

Implementing actions at all three levels or only at one or two levels seemed not to have effect on objectively measured PA or SB changes. This finding is contradictory to the views favoring multilevel approaches in encouraging healthy behaviors in worksite settings [47]. However, to our knowledge, no intervention studies have yet been published, which have compared multilevel and single-level approaches in PA promotion or SB reduction at worksite setting. Thus, more research is needed in this area.

### Strengths

The primary strength of the study was that it was conducted in a real-world setting. This may allow more direct translation of the results to practice and policy [23]. Also, the actions were mostly planned and implemented by the workplaces themselves, which made the intervention highly participative. In a study by Witt et al. [58] involving small and midsize enterprises the employee buy-in was seen the most prominent barrier to health promotion. The companies thus emphasized the role of building employee-ownership to health promotion actions by involving workers in the planning process or surveying their preferences before planning and implementing the health promotion actions. Participatory actions may also enhance the sustainability of behavior changes beyond the intervention period [59].

Another strength pertains to the fact that the implementation of the present intervention followed a theoretical background, particularly multilevel principles of the socio-ecological models, but within the terms of each individual workplace. The novelty and value of the MTB intervention is thus that the selection of the multilevel actions was based on the needs and resources of individual workplaces. However, it is acknowledged that this inevitably led to a wide variety in actions and their contents and delivery, which made it impractical to evaluate which actions or combinations of actions were the ones behind the changes. Nevertheless, according to previous studies [60] it seems unlikely that one specific action or combination of actions would be equally feasible and effective in all workplaces where it is being implemented. It has been suggested that multiple factors [61], for instance organizational climate [62], affect employees’ participation in workplace health promotion and subsequently the feasibility and outcomes of the health promotion activities [63]. In this study the actions were planned and implemented by the MTB teams, who had the best internal knowledge about what was likely to work in their workplaces. This way we expected to maximize employees’ participation in the measurements and also their exposure to the actions.

The present study included also the evaluation of implementation covering both the organization of the MTB teams and the actions carried out in the workplaces. The importance of evaluating the implementation of workplace interventions has been highlighted to understand the intervention effects [64, 65] and to better translate research to practice [66, 67].

One asset of the present study was also the diversity of the size and field of activity of the workplaces involved. This supports the feasibility of similar interventions in various types of workplaces. On the other hand, the diversity prevents one from analyzing changes and making conclusions about the generalizability of the results in certain types of workplaces since the number of similar workplaces, e.g. those with manual workers only, was low.

Finally, the strength of the study was the use of objective measures along with self-reports in assessing PA and SB. Although accelerometers have become common in real-world interventions, their use is still subject to various issues in wearing compliance and data processing issues [53]. In this study, the compliance of the employees was facilitated by providing them with personal feedback from the accelerometer data. The data processing issues were managed by using raw acceleration data and a novel classification method, which is able to distinguish intensity-specific PA and to separate SB and reclining posture from standing. However, as reclined posture could not be reliably separated from sitting, it was combined with sitting. This is in line with the current definition of SB [4].

### Limitations

The study also had some limitations, which should be recognized when interpreting the results. Most notably, the study did not include a control group. Thus, it cannot be exclusively concluded that the positive changes in PA and SB from baseline to follow-up were due to the intervention. It may have been, for example, that the general awareness among the working-aged population about the health hazards of SB increased during the intervention and decreased SB in the workplaces regardless of the intervention. To see the actual effects of the intervention on PA and SB, the study needs to be repeated in a condition, where some workplaces are randomly assigned to the similar intervention as conducted in this study and other workplaces are randomly exposed to measurements only. Unfortunately the financial and human resources allocated to the present study did not allow a controlled design.

Attention should also be paid to the fact that the workplaces participating in the study were interested in developing their practices related to PA promotion and therefore represented a convenience sample of workplaces with high receptivity. The same applies to the employees, who participated in the intervention by responding to the questionnaire and completing the accelerometer and diary measurements. This kind of selectivity happens to some extent in most studies conducted in worksite setting [68] and may not be totally removed because the workplaces and employees cannot be forced to participate due to ethical reasons. In this study the completion rates of the self-reported and objective baseline measurements (74% and 67%, respectively) were higher than the mean adherence in worksite health promotion programs [68]. Furthermore, no difference was detected in the background characteristics, PA and SB between the employees who participated only in the baseline measurements and in those who participated in both measurements. It seems, therefore, that despite the possible selection bias caused by the voluntariness in the beginning of the study, no such selection happened within the participating employees during the course of the intervention, which would have weakened the representativeness of the sample and furthermore the interpretation of the results in this particular sample.

### Comparison with earlier studies

We are not aware of any study, which has applied socio-ecological models in exactly similar way to promote PA and reduce SB in a workplace setting. The randomized controlled 12-week Move to Improve –intervention by Dishman et al. [69] resembles the present intervention. It aimed to increase moderate and vigorous-intensity PA in 16 worksites with 1442 employees by applying goal setting at organizational, team and employee level. The outcomes in PA (SB was not evaluated) were assessed with the short form of International Physical Activity Questionnaire (IPAQ) and a pedometer. As a result, walking and the levels of moderate and vigorous-intensity PA increased in comparison with the control group. Also the proportion of employees meeting the PA recommendations increased. The findings indicate the effectiveness of multilevel approach also in a controlled condition. However, due to different contents of the intervention the results are not comparable with the present study.

Another resemplance to the present MTB-intervention is a cluster randomized Stand Up Victoria trial conducted in the 14 sites of one large government organization in Australia [70]. It targeted at reducing employees’ (*n* = 231) sitting and applied multilevel approach including organizational (management consultation, workshop for the representatives, brainstorming session for the participants), environmental (sit-stand workstation + instructions on ergonomics) and individual (health coaching and supportive telephone calls) strategies. Assessment of sitting was based on 7-day accelerometer-use at baseline and at three and 12 months. Large differences in changes were discovered between the intervention and control sites at both follow-ups in daily workplace sitting (−99 and −45 min) and standing (+95 and +43 min) as well as in daily overall sitting (−78 and −36 min) and standing (+76 and +41 min) [71]. Although the changes in sitting were larger and the procedure and elements of the Stand Up Victoria -intervention were different from MTB, it supports the present findings. Encouraging results have also been obtained from smaller multilevel trials aiming to reduce sitting (e.g. [72]).

In summary, MTB and similar interventions with comparison groups seem to support multilevel approaches to promote PA and reduce SB at worksite setting. However, more research is still needed on the effectiveness of specific actions or combinations of actions implemented at different levels.

## Conclusions

During the MTB intervention, employees’ PA increased and SB reduced at work. Concurrently, employees’ leisure PA slightly decreased but clearly less than the PA at work increased. Number of actions or levels seemed not to have effect on results. Apparently, workplaces can achieve meaningful changes in employees’ PA and SB if assisted systematically. Controlled studies with similar participatory multilevel approach are needed to confirm the present findings.

## Additional files

Additional file 1: Assessment of physical activity (PA), sedentary behavior (SB) and possibility to break sitting in the baseline and follow-up questionnaire to the employees. The questions used in the study questionnaire to quantify physical activity and sedentary behavior. (DOCX 115 kb)

Additional file 2: Statistical analysis to test the baseline differences between the employees, who had completed the questionnaire and the accelerometer measurements only at baseline and who had completed the information also at 1-year follow-up. Statistical analysis. (DOCX 181 kb)

Additional file 3 Statistical analysis to test the difference in change in physical activity and sedentary behavior between the workplaces implementing more or fewer actions than on average and between the workplaces implementing actions at all three levels and just one or two levels. Statistical analysis. (DOCX 105 kb)

## Abbreviations

MAD: Mean amplitude deviation
MTB: Moving to Business -intervention
PA: Physical activity
SB: Sedentary behavior
