# Supplementary material for: Moving to business – changes in physical activity and sedentary behavior after multilevel intervention in small and medium-size workplaces
Source: BMC Public Health. 2017 Apr 17;17:319. doi: 10.1186/s12889-017-4229-4 (PMC5392953; doi:10.1186/s12889-017-4229-4)
Supplement: Supplementary file 1 — Assessment of physical activity (PA), sedentary behavior (SB) and possibility to break sitting in the baseline and follow-up questionnaire to the employees. The questions used in the study questionnaire to quantify physical activity and sedentary behavior. (DOCX 115 kb) [file 12889_2017_4229_MOESM1_ESM.docx]

Additional file 1. Assessment of physical activity (PA), sedentary behavior (SB) and possibility to break sitting in the baseline and follow-up questionnaire to the employees.

| **How much physical activity are you engaged in during a week?** Take into account all weekly regular physical exertion that lasts at least 10 minutes at a time. Circle all alternatives that describe your physical activity and indicate the weekly duration of each particular alternative. If you are not engaged in any weekly regular physical activity, circle “No regular physical activity every week” and leave other alternatives blank. | | | |
| --- | --- | --- | --- |
| 1 | No regular physical activity every week. | | |
| 2 | Light-intensity physical activity (no sweating and/or breathlessness, e.g. slow walking) on ____ days a week totaling ____ hours ____ minutes a week. | | |
| 3 | Moderate-intensity physical activity (some sweating and/or breathlessness, e.g. brisk walking) on ____ days a week totaling ____ hours ____ minutes a week. | | |
| 4 | Vigorous-intensity physical activity (strong sweating and/or breathlessness, e.g. jogging or running) on ____ days a week totaling ____ hours ____ minutes a week. | | |
| 5 | Muscular training (e.g. circular training or fitness training with at least 8 to 12 repetitions for various muscle groups) on ____ days a week totaling ____ hours ____ minutes a week. | | |
| 6 | Balance training (e.g. tai-chi, dancing, exergames, balance exercises on one foot, uneven surface or crawling position) on ____ days a week totaling ____ hours ____ minutes a week. | | |
| **What is the primary transportation mode you currently use for work journeys?** | | | |
| 1 | By foot | | |
| 2 | By bicycle | | |
| 3 | By bus | | |
| 4 | By motorcycle or moped | | |
| 5 | By car | | |
| 6 | By something else | | |
| **In average, how long do you sit on a regular working day?** | | | |
| At work | | _____ hours | _____ minutes a day |
| At home watching TV or videos | | _____ hours | _____ minutes a day |
| At home at the computer | | _____ hours | _____ minutes a day |
| During transportation | | _____ hours | _____ minutes a day |
| Elsewhere | | _____ hours | _____ minutes a day |
| **In average, how long do you sit on a regular non-working day?** | | | |
| At home watching TV or videos | | _____ hours | _____ minutes a day |
| At home at the computer | | _____ hours | _____ minutes a day |
| During transportation | | _____ hours | _____ minutes a day |
| Elsewhere | | _____ hours | _____ minutes a day |
| **What kind of possibility do you have to break work-related sitting by standing or walking?** | | | |
| 1 | Very poor | | |
| 2 | Poor | | |
| 3 | Good | | |
| 4 | Very good | | |
| 5 | I do not sit much at work. | | |
| **How often do you break daily work-related sitting by standing or walking?** | | | |
| 1 | At least once in a half an hour | | |
| 2 | Once in an hour | | |
| 3 | Once in two hours | | |
| 4 | Once in three hours | | |
| 5 | Once in four hours | | |
| 6 | Less than once in four hours or not at all | | |
| 7 | I do not sit much at work. | | |
