# Supplementary material for: Moving to business – changes in physical activity and sedentary behavior after multilevel intervention in small and medium-size workplaces
Source: BMC Public Health. 2017 Apr 17;17:319. doi: 10.1186/s12889-017-4229-4 (PMC5392953; doi:10.1186/s12889-017-4229-4)
Supplement: Supplementary file 2 — Statistical analysis to test the baseline differences between the employees, who had completed the questionnaire and the accelerometer measurements only at baseline and who had completed the information also at 1-year follow-up. Statistical analysis. (DOCX 181 kb) [file 12889_2017_4229_MOESM2_ESM.docx]

Additional file 2: Statistical analysis to test the baseline differences between the employees, who had completed the questionnaire and the accelerometer measurements only at baseline and who had completed the information also at one-year follow-up.

*############### kyselyn taustat ########################.

CROSSTABS

/TABLES=SUKUP BY molemmat_kyselyt

/FORMAT=AVALUE TABLES

/STATISTICS=CHISQ

/CELLS=COUNT COLUMN

/COUNT ROUND CELL.

**Crosstabs**

| **Case Processing Summary** | | | | | | |
| --- | --- | --- | --- | --- | --- | --- |
|  | Cases | | | | | |
|  | Valid | | Missing | | Total | |
|  | N | Percent | N | Percent | N | Percent |
| SUKUP * molemmat_kyselyt | 294 | 91,0% | 29 | 9,0% | 323 | 100,0% |

| **SUKUP * molemmat_kyselyt Crosstabulation** | | | | | |
| --- | --- | --- | --- | --- | --- |
|  | | | molemmat_kyselyt | | Total |
|  |  |  | Vain alkukysely | Alku- ja loppukysely |  |
| SUKUP | 1 | Count | 44 | 62 | 106 |
|  |  | % within molemmat_kyselyt | 40,7% | 33,3% | 36,1% |
|  | 2 | Count | 64 | 124 | 188 |
|  |  | % within molemmat_kyselyt | 59,3% | 66,7% | 63,9% |
| Total | | Count | 108 | 186 | 294 |
|  |  | % within molemmat_kyselyt | 100,0% | 100,0% | 100,0% |

| **Chi-Square Tests** | | | | | |
| --- | --- | --- | --- | --- | --- |
|  | Value | df | Asymp. Sig. (2-sided) | Exact Sig. (2-sided) | Exact Sig. (1-sided) |
| Pearson Chi-Square | 1,626^a^ | 1 | ,202 |  |  |
| Continuity Correction^b^ | 1,321 | 1 | ,250 |  |  |
| Likelihood Ratio | 1,616 | 1 | ,204 |  |  |
| Fisher's Exact Test |  |  |  | ,210 | ,125 |
| Linear-by-Linear Association | 1,621 | 1 | ,203 |  |  |
| N of Valid Cases | 294 |  |  |  |  |
| a. 0 cells (,0%) have expected count less than 5. The minimum expected count is 38,94. | | | | | |
| b. Computed only for a 2x2 table | | | | | |

T-TEST GROUPS=molemmat_kyselyt(0 1)

/MISSING=ANALYSIS

/VARIABLES=IKA

/CRITERIA=CI(.95).

**T-Test**

| **Group Statistics** | | | | | |
| --- | --- | --- | --- | --- | --- |
|  | molemmat_kyselyt | N | Mean | Std. Deviation | Std. Error Mean |
| IKA | Vain alkukysely | 108 | 43,86 | 11,718 | 1,128 |
|  | Alku- ja loppukysely | 186 | 41,72 | 10,355 | ,759 |

| **Independent Samples Test** | | | | | | | | | | |
| --- | --- | --- | --- | --- | --- | --- | --- | --- | --- | --- |
|  | | Levene's Test for Equality of Variances | | t-test for Equality of Means | | | | | | |
|  |  | F | Sig. | t | df | Sig. (2-tailed) | Mean Difference | Std. Error Difference | 95% Confidence Interval of the Difference | |
|  |  |  |  |  |  |  |  |  | Lower | Upper |
| IKA | Equal variances assumed | 3,970 | ,047 | 1,631 | 292 | ,104 | 2,146 | 1,316 | -,443 | 4,735 |
|  | Equal variances not assumed |  |  | 1,579 | 202,011 | ,116 | 2,146 | 1,359 | -,534 | 4,826 |

CROSSTABS

/TABLES=TERVEYS1_3lk BY molemmat_kyselyt

/FORMAT=AVALUE TABLES

/STATISTICS=CHISQ

/CELLS=COUNT COLUMN

/COUNT ROUND CELL.

**Crosstabs**

| **Case Processing Summary** | | | | | | |
| --- | --- | --- | --- | --- | --- | --- |
|  | Cases | | | | | |
|  | Valid | | Missing | | Total | |
|  | N | Percent | N | Percent | N | Percent |
| 3-luokkainen työn terveysdentila * molemmat_kyselyt | 293 | 90,7% | 30 | 9,3% | 323 | 100,0% |

| **3-luokkainen työn terveysdentila * molemmat_kyselyt Crosstabulation** | | | | | |
| --- | --- | --- | --- | --- | --- |
|  | | | molemmat_kyselyt | | Total |
|  |  |  | Vain alkukysely | Alku- ja loppukysely |  |
| 3-luokkainen työn terveysdentila | erittäin huono tai huono | Count | 3 | 7 | 10 |
|  |  | % within molemmat_kyselyt | 2,8% | 3,8% | 3,4% |
|  | keskitasoinen | Count | 33 | 42 | 75 |
|  |  | % within molemmat_kyselyt | 30,6% | 22,7% | 25,6% |
|  | melko hyvä tai hyvä | Count | 72 | 136 | 208 |
|  |  | % within molemmat_kyselyt | 66,7% | 73,5% | 71,0% |
| Total | | Count | 108 | 185 | 293 |
|  |  | % within molemmat_kyselyt | 100,0% | 100,0% | 100,0% |

| **Chi-Square Tests** | | | |
| --- | --- | --- | --- |
|  | Value | df | Asymp. Sig. (2-sided) |
| Pearson Chi-Square | 2,295^a^ | 2 | ,317 |
| Likelihood Ratio | 2,269 | 2 | ,322 |
| Linear-by-Linear Association | ,807 | 1 | ,369 |
| N of Valid Cases | 293 |  |  |
| a. 1 cells (16,7%) have expected count less than 5. The minimum expected count is 3,69. | | | |

CROSSTABS

/TABLES=RUUMRAS_3lk BY molemmat_kyselyt

/FORMAT=AVALUE TABLES

/STATISTICS=CHISQ

/CELLS=COUNT COLUMN

/COUNT ROUND CELL.

**Crosstabs**

| **Case Processing Summary** | | | | | | |
| --- | --- | --- | --- | --- | --- | --- |
|  | Cases | | | | | |
|  | Valid | | Missing | | Total | |
|  | N | Percent | N | Percent | N | Percent |
| 3-luokkainen työn rasittavuus * molemmat_kyselyt | 291 | 90,1% | 32 | 9,9% | 323 | 100,0% |

| **3-luokkainen työn rasittavuus * molemmat_kyselyt Crosstabulation** | | | | | |
| --- | --- | --- | --- | --- | --- |
|  | | | molemmat_kyselyt | | Total |
|  |  |  | Vain alkukysely | Alku- ja loppukysely |  |
| 3-luokkainen työn rasittavuus | luokat 1 ja 2 | Count | 75 | 130 | 205 |
|  |  | % within molemmat_kyselyt | 69,4% | 71,0% | 70,4% |
|  | luokat 3 ja 4 | Count | 27 | 42 | 69 |
|  |  | % within molemmat_kyselyt | 25,0% | 23,0% | 23,7% |
|  | luokat 5 ja 6 | Count | 6 | 11 | 17 |
|  |  | % within molemmat_kyselyt | 5,6% | 6,0% | 5,8% |
| Total | | Count | 108 | 183 | 291 |
|  |  | % within molemmat_kyselyt | 100,0% | 100,0% | 100,0% |

| **Chi-Square Tests** | | | |
| --- | --- | --- | --- |
|  | Value | df | Asymp. Sig. (2-sided) |
| Pearson Chi-Square | ,169^a^ | 2 | ,919 |
| Likelihood Ratio | ,168 | 2 | ,919 |
| Linear-by-Linear Association | ,025 | 1 | ,873 |
| N of Valid Cases | 291 |  |  |
| a. 0 cells (,0%) have expected count less than 5. The minimum expected count is 6,31. | | | |

CROSSTABS

/TABLES= TYOAIKA_3lk BY molemmat_kyselyt

/FORMAT=AVALUE TABLES

/STATISTICS=CHISQ

/CELLS=COUNT COLUMN

/COUNT ROUND CELL.

**Crosstabs**

| **Case Processing Summary** | | | | | | |
| --- | --- | --- | --- | --- | --- | --- |
|  | Cases | | | | | |
|  | Valid | | Missing | | Total | |
|  | N | Percent | N | Percent | N | Percent |
| 3-luokkainen tyoaika * molemmat_kyselyt | 293 | 90,7% | 30 | 9,3% | 323 | 100,0% |

| **3-luokkainen tyoaika * molemmat_kyselyt Crosstabulation** | | | | | |
| --- | --- | --- | --- | --- | --- |
|  | | | molemmat_kyselyt | | Total |
|  |  |  | Vain alkukysely | Alku- ja loppukysely |  |
| 3-luokkainen tyoaika | 1,00 | Count | 81 | 135 | 216 |
|  |  | % within molemmat_kyselyt | 75,0% | 73,0% | 73,7% |
|  | 2,00 | Count | 8 | 14 | 22 |
|  |  | % within molemmat_kyselyt | 7,4% | 7,6% | 7,5% |
|  | 3,00 | Count | 19 | 36 | 55 |
|  |  | % within molemmat_kyselyt | 17,6% | 19,5% | 18,8% |
| Total | | Count | 108 | 185 | 293 |
|  |  | % within molemmat_kyselyt | 100,0% | 100,0% | 100,0% |

| **Chi-Square Tests** | | | |
| --- | --- | --- | --- |
|  | Value | df | Asymp. Sig. (2-sided) |
| Pearson Chi-Square | ,167^a^ | 2 | ,920 |
| Likelihood Ratio | ,168 | 2 | ,919 |
| Linear-by-Linear Association | ,165 | 1 | ,684 |
| N of Valid Cases | 293 |  |  |
| a. 0 cells (,0%) have expected count less than 5. The minimum expected count is 8,11. | | | |

CROSSTABS

/TABLES= KOUL_3lk BY molemmat_kyselyt

/FORMAT=AVALUE TABLES

/STATISTICS=CHISQ

/CELLS=COUNT COLUMN

/COUNT ROUND CELL.

**Crosstabs**

| **Case Processing Summary** | | | | | | |
| --- | --- | --- | --- | --- | --- | --- |
|  | Cases | | | | | |
|  | Valid | | Missing | | Total | |
|  | N | Percent | N | Percent | N | Percent |
| 3-luokkainen koulutus * molemmat_kyselyt | 292 | 90,4% | 31 | 9,6% | 323 | 100,0% |

| **3-luokkainen koulutus * molemmat_kyselyt Crosstabulation** | | | | | |
| --- | --- | --- | --- | --- | --- |
|  | | | molemmat_kyselyt | | Total |
|  |  |  | Vain alkukysely | Alku- ja loppukysely |  |
| 3-luokkainen koulutus | luokat 1-3 | Count | 32 | 43 | 75 |
|  |  | % within molemmat_kyselyt | 29,6% | 23,4% | 25,7% |
|  | luokka 4 | Count | 53 | 85 | 138 |
|  |  | % within molemmat_kyselyt | 49,1% | 46,2% | 47,3% |
|  | luokat 5 ja 6 | Count | 23 | 56 | 79 |
|  |  | % within molemmat_kyselyt | 21,3% | 30,4% | 27,1% |
| Total | | Count | 108 | 184 | 292 |
|  |  | % within molemmat_kyselyt | 100,0% | 100,0% | 100,0% |

| **Chi-Square Tests** | | | |
| --- | --- | --- | --- |
|  | Value | df | Asymp. Sig. (2-sided) |
| Pearson Chi-Square | 3,258^a^ | 2 | ,196 |
| Likelihood Ratio | 3,314 | 2 | ,191 |
| Linear-by-Linear Association | 3,050 | 1 | ,081 |
| N of Valid Cases | 292 |  |  |
| a. 0 cells (,0%) have expected count less than 5. The minimum expected count is 27,74. | | | |

*################# kyselyn outcomet #####.

T-TEST GROUPS=molemmat_kyselyt(0 1)

/MISSING=ANALYSIS

/VARIABLES=LIIKKESTO1

/CRITERIA=CI(.95).

**T-Test**

| **Group Statistics** | | | | | |
| --- | --- | --- | --- | --- | --- |
|  | molemmat_kyselyt | N | Mean | Std. Deviation | Std. Error Mean |
| Liikkumisen kesto minuuteissa yhteensä | Vain alkukysely | 108 | 247,2500 | 246,35440 | 23,70546 |
|  | Alku- ja loppukysely | 185 | 254,7892 | 284,55374 | 20,92081 |

| **Independent Samples Test** | | | | | | | | | | |
| --- | --- | --- | --- | --- | --- | --- | --- | --- | --- | --- |
|  | | Levene's Test for Equality of Variances | | t-test for Equality of Means | | | | | | |
|  |  | F | Sig. | t | df | Sig. (2-tailed) | Mean Difference | Std. Error Difference | 95% Confidence Interval of the Difference | |
|  |  |  |  |  |  |  |  |  | Lower | Upper |
| Liikkumisen kesto minuuteissa yhteensä | Equal variances assumed | ,037 | ,847 | -,230 | 291 | ,819 | -7,53919 | 32,83375 | -72,16092 | 57,08254 |
|  | Equal variances not assumed |  |  | -,238 | 250,291 | ,812 | -7,53919 | 31,61691 | -69,80829 | 54,72992 |

T-TEST GROUPS=molemmat_kyselyt(0 1)

/MISSING=ANALYSIS

/VARIABLES=VERKKESTO1

/CRITERIA=CI(.95).

**T-Test**

| **Group Statistics** | | | | | |
| --- | --- | --- | --- | --- | --- |
|  | molemmat_kyselyt | N | Mean | Std. Deviation | Std. Error Mean |
| Verkkaisen liikkumisen viikoittainen kesto minuuteissa | Vain alkukysely | 108 | 100,8611 | 154,89879 | 14,90514 |
|  | Alku- ja loppukysely | 185 | 100,0000 | 240,52535 | 17,68378 |

| **Independent Samples Test** | | | | | | | | | | |
| --- | --- | --- | --- | --- | --- | --- | --- | --- | --- | --- |
|  | | Levene's Test for Equality of Variances | | t-test for Equality of Means | | | | | | |
|  |  | F | Sig. | t | df | Sig. (2-tailed) | Mean Difference | Std. Error Difference | 95% Confidence Interval of the Difference | |
|  |  |  |  |  |  |  |  |  | Lower | Upper |
| Verkkaisen liikkumisen viikoittainen kesto minuuteissa | Equal variances assumed | ,314 | ,576 | ,033 | 291 | ,973 | ,86111 | 25,80338 | -49,92380 | 51,64603 |
|  | Equal variances not assumed |  |  | ,037 | 288,185 | ,970 | ,86111 | 23,12746 | -44,65904 | 46,38126 |

T-TEST GROUPS=molemmat_kyselyt(0 1)

/MISSING=ANALYSIS

/VARIABLES=REIKESTO1

/CRITERIA=CI(.95).

**T-Test**

| **Group Statistics** | | | | | |
| --- | --- | --- | --- | --- | --- |
|  | molemmat_kyselyt | N | Mean | Std. Deviation | Std. Error Mean |
| Reippaan liikkumisen viikoittainen kesto minuuteissa | Vain alkukysely | 108 | 95,9259 | 166,64943 | 16,03585 |
|  | Alku- ja loppukysely | 185 | 107,7351 | 159,75239 | 11,74523 |

| **Independent Samples Test** | | | | | | | | | | |
| --- | --- | --- | --- | --- | --- | --- | --- | --- | --- | --- |
|  | | Levene's Test for Equality of Variances | | t-test for Equality of Means | | | | | | |
|  |  | F | Sig. | t | df | Sig. (2-tailed) | Mean Difference | Std. Error Difference | 95% Confidence Interval of the Difference | |
|  |  |  |  |  |  |  |  |  | Lower | Upper |
| Reippaan liikkumisen viikoittainen kesto minuuteissa | Equal variances assumed | ,255 | ,614 | -,601 | 291 | ,548 | -11,80921 | 19,65689 | -50,49690 | 26,87849 |
|  | Equal variances not assumed |  |  | -,594 | 216,383 | ,553 | -11,80921 | 19,87709 | -50,98672 | 27,36830 |

T-TEST GROUPS=molemmat_kyselyt(0 1)

/MISSING=ANALYSIS

/VARIABLES=RASKESTO1

/CRITERIA=CI(.95).

**T-Test**

| **Group Statistics** | | | | | |
| --- | --- | --- | --- | --- | --- |
|  | molemmat_kyselyt | N | Mean | Std. Deviation | Std. Error Mean |
| Rasittavan liikkumisen viikoittaine kesto minuuteissa | Vain alkukysely | 108 | 50,4630 | 79,87589 | 7,68606 |
|  | Alku- ja loppukysely | 185 | 47,0541 | 73,61103 | 5,41199 |

| **Independent Samples Test** | | | | | | | | | | |
| --- | --- | --- | --- | --- | --- | --- | --- | --- | --- | --- |
|  | | Levene's Test for Equality of Variances | | t-test for Equality of Means | | | | | | |
|  |  | F | Sig. | t | df | Sig. (2-tailed) | Mean Difference | Std. Error Difference | 95% Confidence Interval of the Difference | |
|  |  |  |  |  |  |  |  |  | Lower | Upper |
| Rasittavan liikkumisen viikoittaine kesto minuuteissa | Equal variances assumed | ,727 | ,394 | ,371 | 291 | ,711 | 3,40891 | 9,20036 | -14,69878 | 21,51660 |
|  | Equal variances not assumed |  |  | ,363 | 209,462 | ,717 | 3,40891 | 9,40028 | -15,12236 | 21,94018 |

T-TEST GROUPS=molemmat_kyselyt(0 1)

/MISSING=ANALYSIS

/VARIABLES=TIST_YHTPVA1

/CRITERIA=CI(.95).

**T-Test**

| **Group Statistics** | | | | | |
| --- | --- | --- | --- | --- | --- |
|  | molemmat_kyselyt | N | Mean | Std. Deviation | Std. Error Mean |
| Työssäkäyntipäivä, päivittäinen istuminen minuuteissa yhteensä | Vain alkukysely | 106 | 502,2170 | 152,02613 | 14,76608 |
|  | Alku- ja loppukysely | 184 | 511,8641 | 137,08040 | 10,10570 |

| **Independent Samples Test** | | | | | | | | | | |
| --- | --- | --- | --- | --- | --- | --- | --- | --- | --- | --- |
|  | | Levene's Test for Equality of Variances | | t-test for Equality of Means | | | | | | |
|  |  | F | Sig. | t | df | Sig. (2-tailed) | Mean Difference | Std. Error Difference | 95% Confidence Interval of the Difference | |
|  |  |  |  |  |  |  |  |  | Lower | Upper |
| Työssäkäyntipäivä, päivittäinen istuminen minuuteissa yhteensä | Equal variances assumed | 2,005 | ,158 | -,554 | 288 | ,580 | -9,64715 | 17,40179 | -43,89797 | 24,60367 |
|  | Equal variances not assumed |  |  | -,539 | 201,084 | ,590 | -9,64715 | 17,89308 | -44,92929 | 25,63499 |

T-TEST GROUPS=molemmat_kyselyt(0 1)

/MISSING=ANALYSIS

/VARIABLES=TIST_TYOKESTO1

/CRITERIA=CI(.95).

**T-Test**

| **Group Statistics** | | | | | |
| --- | --- | --- | --- | --- | --- |
|  | molemmat_kyselyt | N | Mean | Std. Deviation | Std. Error Mean |
| Työssäkäyntipäivä, päivittäinen istuminen minuuteissa töissä | Vain alkukysely | 106 | 302,5472 | 145,01764 | 14,08536 |
|  | Alku- ja loppukysely | 184 | 305,3696 | 138,39614 | 10,20270 |

| **Independent Samples Test** | | | | | | | | | | |
| --- | --- | --- | --- | --- | --- | --- | --- | --- | --- | --- |
|  | | Levene's Test for Equality of Variances | | t-test for Equality of Means | | | | | | |
|  |  | F | Sig. | t | df | Sig. (2-tailed) | Mean Difference | Std. Error Difference | 95% Confidence Interval of the Difference | |
|  |  |  |  |  |  |  |  |  | Lower | Upper |
| Työssäkäyntipäivä, päivittäinen istuminen minuuteissa töissä | Equal variances assumed | ,693 | ,406 | -,164 | 288 | ,870 | -2,82240 | 17,17444 | -36,62573 | 30,98094 |
|  | Equal variances not assumed |  |  | -,162 | 210,793 | ,871 | -2,82240 | 17,39231 | -37,10754 | 31,46275 |

T-TEST GROUPS=molemmat_kyselyt(0 1)

/MISSING=ANALYSIS

/VARIABLES=TIST_KULKESTO1

/CRITERIA=CI(.95).

**T-Test**

| **Group Statistics** | | | | | |
| --- | --- | --- | --- | --- | --- |
|  | molemmat_kyselyt | N | Mean | Std. Deviation | Std. Error Mean |
| Työssäkäyntipäivä, päivittäinen istuminen minuuteissa kulkuneuvossa | Vain alkukysely | 106 | 44,1981 | 41,74832 | 4,05496 |
|  | Alku- ja loppukysely | 184 | 38,3696 | 40,00757 | 2,94940 |

| **Independent Samples Test** | | | | | | | | | | |
| --- | --- | --- | --- | --- | --- | --- | --- | --- | --- | --- |
|  | | Levene's Test for Equality of Variances | | t-test for Equality of Means | | | | | | |
|  |  | F | Sig. | t | df | Sig. (2-tailed) | Mean Difference | Std. Error Difference | 95% Confidence Interval of the Difference | |
|  |  |  |  |  |  |  |  |  | Lower | Upper |
| Työssäkäyntipäivä, päivittäinen istuminen minuuteissa kulkuneuvossa | Equal variances assumed | ,810 | ,369 | 1,176 | 288 | ,241 | 5,82855 | 4,95686 | -3,92772 | 15,58482 |
|  | Equal variances not assumed |  |  | 1,162 | 211,520 | ,246 | 5,82855 | 5,01414 | -4,05554 | 15,71264 |

*########################## HOOKIE ##########################.

*############### kyselyn taustat ########################.

CROSSTABS

/TABLES=SUKUP BY molemmat_hookiet

/FORMAT=AVALUE TABLES

/STATISTICS=CHISQ

/CELLS=COUNT COLUMN

/COUNT ROUND CELL.

**Crosstabs**

| **Case Processing Summary** | | | | | | |
| --- | --- | --- | --- | --- | --- | --- |
|  | Cases | | | | | |
|  | Valid | | Missing | | Total | |
|  | N | Percent | N | Percent | N | Percent |
| SUKUP * molemmat_hookiet | 254 | 78,6% | 69 | 21,4% | 323 | 100,0% |

| **SUKUP * molemmat_hookiet Crosstabulation** | | | | | |
| --- | --- | --- | --- | --- | --- |
|  | | | molemmat_hookiet | | Total |
|  |  |  | Hookie vain alussa 3 pv 10h | Hookie alussa ja lopussa 3 pv 10h |  |
| SUKUP | 1 | Count | 42 | 45 | 87 |
|  |  | % within molemmat_hookiet | 37,5% | 31,7% | 34,3% |
|  | 2 | Count | 70 | 97 | 167 |
|  |  | % within molemmat_hookiet | 62,5% | 68,3% | 65,7% |
| Total | | Count | 112 | 142 | 254 |
|  |  | % within molemmat_hookiet | 100,0% | 100,0% | 100,0% |

| **Chi-Square Tests** | | | | | |
| --- | --- | --- | --- | --- | --- |
|  | Value | df | Asymp. Sig. (2-sided) | Exact Sig. (2-sided) | Exact Sig. (1-sided) |
| Pearson Chi-Square | ,939^a^ | 1 | ,333 |  |  |
| Continuity Correction^b^ | ,698 | 1 | ,403 |  |  |
| Likelihood Ratio | ,936 | 1 | ,333 |  |  |
| Fisher's Exact Test |  |  |  | ,353 | ,202 |
| Linear-by-Linear Association | ,935 | 1 | ,334 |  |  |
| N of Valid Cases | 254 |  |  |  |  |
| a. 0 cells (,0%) have expected count less than 5. The minimum expected count is 38,36. | | | | | |
| b. Computed only for a 2x2 table | | | | | |

T-TEST GROUPS=molemmat_hookiet(0 1)

/MISSING=ANALYSIS

/VARIABLES=IKA

/CRITERIA=CI(.95).

**T-Test**

| **Group Statistics** | | | | | |
| --- | --- | --- | --- | --- | --- |
|  | molemmat_hookiet | N | Mean | Std. Deviation | Std. Error Mean |
| IKA | Hookie vain alussa 3 pv 10h | 112 | 42,40 | 11,099 | 1,049 |
|  | Hookie alussa ja lopussa 3 pv 10h | 142 | 42,49 | 10,774 | ,904 |

| **Independent Samples Test** | | | | | | | | | | |
| --- | --- | --- | --- | --- | --- | --- | --- | --- | --- | --- |
|  | | Levene's Test for Equality of Variances | | t-test for Equality of Means | | | | | | |
|  |  | F | Sig. | t | df | Sig. (2-tailed) | Mean Difference | Std. Error Difference | 95% Confidence Interval of the Difference | |
|  |  |  |  |  |  |  |  |  | Lower | Upper |
| IKA | Equal variances assumed | ,099 | ,753 | -,061 | 252 | ,951 | -,084 | 1,380 | -2,802 | 2,633 |
|  | Equal variances not assumed |  |  | -,061 | 235,088 | ,952 | -,084 | 1,385 | -2,812 | 2,644 |

CROSSTABS

/TABLES=TERVEYS1_3lk BY molemmat_hookiet

/FORMAT=AVALUE TABLES

/STATISTICS=CHISQ

/CELLS=COUNT COLUMN

/COUNT ROUND CELL.

**Crosstabs**

| **Case Processing Summary** | | | | | | |
| --- | --- | --- | --- | --- | --- | --- |
|  | Cases | | | | | |
|  | Valid | | Missing | | Total | |
|  | N | Percent | N | Percent | N | Percent |
| 3-luokkainen työn terveysdentila * molemmat_hookiet | 251 | 77,7% | 72 | 22,3% | 323 | 100,0% |

| **3-luokkainen työn terveysdentila * molemmat_hookiet Crosstabulation** | | | | | |
| --- | --- | --- | --- | --- | --- |
|  | | | molemmat_hookiet | | Total |
|  |  |  | Hookie vain alussa 3 pv 10h | Hookie alussa ja lopussa 3 pv 10h |  |
| 3-luokkainen työn terveysdentila | erittäin huono tai huono | Count | 3 | 3 | 6 |
|  |  | % within molemmat_hookiet | 2,7% | 2,1% | 2,4% |
|  | keskitasoinen | Count | 27 | 33 | 60 |
|  |  | % within molemmat_hookiet | 24,5% | 23,4% | 23,9% |
|  | melko hyvä tai hyvä | Count | 80 | 105 | 185 |
|  |  | % within molemmat_hookiet | 72,7% | 74,5% | 73,7% |
| Total | | Count | 110 | 141 | 251 |
|  |  | % within molemmat_hookiet | 100,0% | 100,0% | 100,0% |

| **Chi-Square Tests** | | | |
| --- | --- | --- | --- |
|  | Value | df | Asymp. Sig. (2-sided) |
| Pearson Chi-Square | ,152^a^ | 2 | ,927 |
| Likelihood Ratio | ,151 | 2 | ,927 |
| Linear-by-Linear Association | ,134 | 1 | ,715 |
| N of Valid Cases | 251 |  |  |
| a. 2 cells (33,3%) have expected count less than 5. The minimum expected count is 2,63. | | | |

CROSSTABS

/TABLES=RUUMRAS_3lk BY molemmat_hookiet

/FORMAT=AVALUE TABLES

/STATISTICS=CHISQ

/CELLS=COUNT COLUMN

/COUNT ROUND CELL.

**Crosstabs**

| **Case Processing Summary** | | | | | | |
| --- | --- | --- | --- | --- | --- | --- |
|  | Cases | | | | | |
|  | Valid | | Missing | | Total | |
|  | N | Percent | N | Percent | N | Percent |
| 3-luokkainen työn rasittavuus * molemmat_hookiet | 251 | 77,7% | 72 | 22,3% | 323 | 100,0% |

| **3-luokkainen työn rasittavuus * molemmat_hookiet Crosstabulation** | | | | | |
| --- | --- | --- | --- | --- | --- |
|  | | | molemmat_hookiet | | Total |
|  |  |  | Hookie vain alussa 3 pv 10h | Hookie alussa ja lopussa 3 pv 10h |  |
| 3-luokkainen työn rasittavuus | luokat 1 ja 2 | Count | 78 | 102 | 180 |
|  |  | % within molemmat_hookiet | 70,9% | 72,3% | 71,7% |
|  | luokat 3 ja 4 | Count | 27 | 31 | 58 |
|  |  | % within molemmat_hookiet | 24,5% | 22,0% | 23,1% |
|  | luokat 5 ja 6 | Count | 5 | 8 | 13 |
|  |  | % within molemmat_hookiet | 4,5% | 5,7% | 5,2% |
| Total | | Count | 110 | 141 | 251 |
|  |  | % within molemmat_hookiet | 100,0% | 100,0% | 100,0% |

| **Chi-Square Tests** | | | |
| --- | --- | --- | --- |
|  | Value | df | Asymp. Sig. (2-sided) |
| Pearson Chi-Square | ,345^a^ | 2 | ,842 |
| Likelihood Ratio | ,346 | 2 | ,841 |
| Linear-by-Linear Association | ,002 | 1 | ,967 |
| N of Valid Cases | 251 |  |  |
| a. 0 cells (,0%) have expected count less than 5. The minimum expected count is 5,70. | | | |

CROSSTABS

/TABLES= TYOAIKA_3lk BY molemmat_hookiet

/FORMAT=AVALUE TABLES

/STATISTICS=CHISQ

/CELLS=COUNT COLUMN

/COUNT ROUND CELL.

**Crosstabs**

| **Case Processing Summary** | | | | | | |
| --- | --- | --- | --- | --- | --- | --- |
|  | Cases | | | | | |
|  | Valid | | Missing | | Total | |
|  | N | Percent | N | Percent | N | Percent |
| 3-luokkainen tyoaika * molemmat_hookiet | 253 | 78,3% | 70 | 21,7% | 323 | 100,0% |

| **3-luokkainen tyoaika * molemmat_hookiet Crosstabulation** | | | | | |
| --- | --- | --- | --- | --- | --- |
|  | | | molemmat_hookiet | | Total |
|  |  |  | Hookie vain alussa 3 pv 10h | Hookie alussa ja lopussa 3 pv 10h |  |
| 3-luokkainen tyoaika | 1,00 | Count | 82 | 105 | 187 |
|  |  | % within molemmat_hookiet | 73,2% | 74,5% | 73,9% |
|  | 2,00 | Count | 5 | 13 | 18 |
|  |  | % within molemmat_hookiet | 4,5% | 9,2% | 7,1% |
|  | 3,00 | Count | 25 | 23 | 48 |
|  |  | % within molemmat_hookiet | 22,3% | 16,3% | 19,0% |
| Total | | Count | 112 | 141 | 253 |
|  |  | % within molemmat_hookiet | 100,0% | 100,0% | 100,0% |

| **Chi-Square Tests** | | | |
| --- | --- | --- | --- |
|  | Value | df | Asymp. Sig. (2-sided) |
| Pearson Chi-Square | 3,186^a^ | 2 | ,203 |
| Likelihood Ratio | 3,271 | 2 | ,195 |
| Linear-by-Linear Association | ,523 | 1 | ,470 |
| N of Valid Cases | 253 |  |  |
| a. 0 cells (,0%) have expected count less than 5. The minimum expected count is 7,97. | | | |

CROSSTABS

/TABLES= KOUL_3lk BY molemmat_hookiet

/FORMAT=AVALUE TABLES

/STATISTICS=CHISQ

/CELLS=COUNT COLUMN

/COUNT ROUND CELL.

**Crosstabs**

| **Case Processing Summary** | | | | | | |
| --- | --- | --- | --- | --- | --- | --- |
|  | Cases | | | | | |
|  | Valid | | Missing | | Total | |
|  | N | Percent | N | Percent | N | Percent |
| 3-luokkainen koulutus * molemmat_hookiet | 252 | 78,0% | 71 | 22,0% | 323 | 100,0% |

| **3-luokkainen koulutus * molemmat_hookiet Crosstabulation** | | | | | |
| --- | --- | --- | --- | --- | --- |
|  | | | molemmat_hookiet | | Total |
|  |  |  | Hookie vain alussa 3 pv 10h | Hookie alussa ja lopussa 3 pv 10h |  |
| 3-luokkainen koulutus | luokat 1-3 | Count | 28 | 32 | 60 |
|  |  | % within molemmat_hookiet | 25,2% | 22,7% | 23,8% |
|  | luokka 4 | Count | 54 | 71 | 125 |
|  |  | % within molemmat_hookiet | 48,6% | 50,4% | 49,6% |
|  | luokat 5 ja 6 | Count | 29 | 38 | 67 |
|  |  | % within molemmat_hookiet | 26,1% | 27,0% | 26,6% |
| Total | | Count | 111 | 141 | 252 |
|  |  | % within molemmat_hookiet | 100,0% | 100,0% | 100,0% |

| **Chi-Square Tests** | | | |
| --- | --- | --- | --- |
|  | Value | df | Asymp. Sig. (2-sided) |
| Pearson Chi-Square | ,219^a^ | 2 | ,896 |
| Likelihood Ratio | ,219 | 2 | ,896 |
| Linear-by-Linear Association | ,138 | 1 | ,710 |
| N of Valid Cases | 252 |  |  |
| a. 0 cells (,0%) have expected count less than 5. The minimum expected count is 26,43. | | | |

*################# Hookien outcomet #####.

T-TEST GROUPS=molemmat_hookiet(0 1)

/MISSING=ANALYSIS

/VARIABLES=LiikkTyo_ka.1

/CRITERIA=CI(.95).

**T-Test**

| **Group Statistics** | | | | | |
| --- | --- | --- | --- | --- | --- |
|  | molemmat_hookiet | N | Mean | Std. Deviation | Std. Error Mean |
| LiikkTyo_ka.1 | Hookie vain alussa 3 pv 10h | 119 | 108,8219 | 67,94246 | 6,22828 |
|  | Hookie alussa ja lopussa 3 pv 10h | 147 | 112,2054 | 79,48450 | 6,55577 |

| **Independent Samples Test** | | | | | | | | | | |
| --- | --- | --- | --- | --- | --- | --- | --- | --- | --- | --- |
|  | | Levene's Test for Equality of Variances | | t-test for Equality of Means | | | | | | |
|  |  | F | Sig. | t | df | Sig. (2-tailed) | Mean Difference | Std. Error Difference | 95% Confidence Interval of the Difference | |
|  |  |  |  |  |  |  |  |  | Lower | Upper |
| LiikkTyo_ka.1 | Equal variances assumed | 1,889 | ,171 | -,368 | 264 | ,713 | -3,38348 | 9,19258 | -21,48358 | 14,71662 |
|  | Equal variances not assumed |  |  | -,374 | 263,199 | ,709 | -3,38348 | 9,04265 | -21,18862 | 14,42167 |

T-TEST GROUPS=molemmat_hookiet(0 1)

/MISSING=ANALYSIS

/VARIABLES=AskelJaJuoksuTyo_ka.1

/CRITERIA=CI(.95).

**T-Test**

| **Group Statistics** | | | | | |
| --- | --- | --- | --- | --- | --- |
|  | molemmat_hookiet | N | Mean | Std. Deviation | Std. Error Mean |
| AskelJaJuoksuTyo_ka.1 | Hookie vain alussa 3 pv 10h | 119 | 3855,7732 | 2265,77095 | 207,70288 |
|  | Hookie alussa ja lopussa 3 pv 10h | 147 | 3758,0603 | 2470,75807 | 203,78469 |

| **Independent Samples Test** | | | | | | | | | | |
| --- | --- | --- | --- | --- | --- | --- | --- | --- | --- | --- |
|  | | Levene's Test for Equality of Variances | | t-test for Equality of Means | | | | | | |
|  |  | F | Sig. | t | df | Sig. (2-tailed) | Mean Difference | Std. Error Difference | 95% Confidence Interval of the Difference | |
|  |  |  |  |  |  |  |  |  | Lower | Upper |
| AskelJaJuoksuTyo_ka.1 | Equal variances assumed | ,352 | ,554 | ,333 | 264 | ,740 | 97,71289 | 293,64720 | -480,47566 | 675,90145 |
|  | Equal variances not assumed |  |  | ,336 | 259,886 | ,737 | 97,71289 | 290,97884 | -475,26345 | 670,68924 |

T-TEST GROUPS=molemmat_hookiet(0 1)

/MISSING=ANALYSIS

/VARIABLES=KevytTyo_ka.1

/CRITERIA=CI(.95).

**T-Test**

| **Group Statistics** | | | | | |
| --- | --- | --- | --- | --- | --- |
|  | molemmat_hookiet | N | Mean | Std. Deviation | Std. Error Mean |
| KevytTyo_ka.1 | Hookie vain alussa 3 pv 10h | 119 | 87,2505 | 55,54751 | 5,09203 |
|  | Hookie alussa ja lopussa 3 pv 10h | 147 | 91,6906 | 67,17860 | 5,54080 |

| **Independent Samples Test** | | | | | | | | | | |
| --- | --- | --- | --- | --- | --- | --- | --- | --- | --- | --- |
|  | | Levene's Test for Equality of Variances | | t-test for Equality of Means | | | | | | |
|  |  | F | Sig. | t | df | Sig. (2-tailed) | Mean Difference | Std. Error Difference | 95% Confidence Interval of the Difference | |
|  |  |  |  |  |  |  |  |  | Lower | Upper |
| KevytTyo_ka.1 | Equal variances assumed | 2,620 | ,107 | -,578 | 264 | ,563 | -4,44018 | 7,67611 | -19,55437 | 10,67401 |
|  | Equal variances not assumed |  |  | -,590 | 263,873 | ,556 | -4,44018 | 7,52524 | -19,25734 | 10,37698 |

T-TEST GROUPS=molemmat_hookiet(0 1)

/MISSING=ANALYSIS

/VARIABLES=ReRaTyo_ka.1

/CRITERIA=CI(.95).

**T-Test**

| **Group Statistics** | | | | | |
| --- | --- | --- | --- | --- | --- |
|  | molemmat_hookiet | N | Mean | Std. Deviation | Std. Error Mean |
| ReRaTyo_ka.1 | Hookie vain alussa 3 pv 10h | 119 | 21,5714 | 18,22230 | 1,67044 |
|  | Hookie alussa ja lopussa 3 pv 10h | 147 | 20,5147 | 17,39894 | 1,43504 |

| **Independent Samples Test** | | | | | | | | | | |
| --- | --- | --- | --- | --- | --- | --- | --- | --- | --- | --- |
|  | | Levene's Test for Equality of Variances | | t-test for Equality of Means | | | | | | |
|  |  | F | Sig. | t | df | Sig. (2-tailed) | Mean Difference | Std. Error Difference | 95% Confidence Interval of the Difference | |
|  |  |  |  |  |  |  |  |  | Lower | Upper |
| ReRaTyo_ka.1 | Equal variances assumed | ,236 | ,627 | ,482 | 264 | ,630 | 1,05670 | 2,19148 | -3,25830 | 5,37170 |
|  | Equal variances not assumed |  |  | ,480 | 247,494 | ,632 | 1,05670 | 2,20220 | -3,28075 | 5,39415 |

T-TEST GROUPS=molemmat_hookiet(0 1)

/MISSING=ANALYSIS

/VARIABLES=IstTyo_ka.1

/CRITERIA=CI(.95).

**T-Test**

| **Group Statistics** | | | | | |
| --- | --- | --- | --- | --- | --- |
|  | molemmat_hookiet | N | Mean | Std. Deviation | Std. Error Mean |
| IstTyo_ka.1 | Hookie vain alussa 3 pv 10h | 119 | 287,8078 | 79,17389 | 7,25786 |
|  | Hookie alussa ja lopussa 3 pv 10h | 147 | 293,5879 | 82,63818 | 6,81588 |

| **Independent Samples Test** | | | | | | | | | | |
| --- | --- | --- | --- | --- | --- | --- | --- | --- | --- | --- |
|  | | Levene's Test for Equality of Variances | | t-test for Equality of Means | | | | | | |
|  |  | F | Sig. | t | df | Sig. (2-tailed) | Mean Difference | Std. Error Difference | 95% Confidence Interval of the Difference | |
|  |  |  |  |  |  |  |  |  | Lower | Upper |
| IstTyo_ka.1 | Equal variances assumed | ,073 | ,787 | -,578 | 264 | ,564 | -5,78002 | 10,00167 | -25,47322 | 13,91317 |
|  | Equal variances not assumed |  |  | -,581 | 256,605 | ,562 | -5,78002 | 9,95654 | -25,38697 | 13,82692 |

T-TEST GROUPS=molemmat_hookiet(0 1)

/MISSING=ANALYSIS

/VARIABLES=Ylos2Tyo_ka.1

/CRITERIA=CI(.95).

**T-Test**

| **Group Statistics** | | | | | |
| --- | --- | --- | --- | --- | --- |
|  | molemmat_hookiet | N | Mean | Std. Deviation | Std. Error Mean |
| Ylos2Tyo_ka.1 | Hookie vain alussa 3 pv 10h | 119 | 23,4063 | 9,25517 | ,84842 |
|  | Hookie alussa ja lopussa 3 pv 10h | 147 | 24,6311 | 9,75965 | ,80496 |

| **Independent Samples Test** | | | | | | | | | | |
| --- | --- | --- | --- | --- | --- | --- | --- | --- | --- | --- |
|  | | Levene's Test for Equality of Variances | | t-test for Equality of Means | | | | | | |
|  |  | F | Sig. | t | df | Sig. (2-tailed) | Mean Difference | Std. Error Difference | 95% Confidence Interval of the Difference | |
|  |  |  |  |  |  |  |  |  | Lower | Upper |
| Ylos2Tyo_ka.1 | Equal variances assumed | 2,216 | ,138 | -1,041 | 264 | ,299 | -1,22476 | 1,17609 | -3,54048 | 1,09095 |
|  | Equal variances not assumed |  |  | -1,047 | 257,451 | ,296 | -1,22476 | 1,16952 | -3,52781 | 1,07828 |

T-TEST GROUPS=molemmat_hookiet(0 1)

/MISSING=ANALYSIS

/VARIABLES=LiikkTyopVA_ka.1

/CRITERIA=CI(.95).

**T-Test**

| **Group Statistics** | | | | | |
| --- | --- | --- | --- | --- | --- |
|  | molemmat_hookiet | N | Mean | Std. Deviation | Std. Error Mean |
| LiikkTyopVA_ka.1 | Hookie vain alussa 3 pv 10h | 119 | 124,4158 | 40,95204 | 3,75407 |
|  | Hookie alussa ja lopussa 3 pv 10h | 147 | 128,3171 | 42,33589 | 3,49181 |

| **Independent Samples Test** | | | | | | | | | | |
| --- | --- | --- | --- | --- | --- | --- | --- | --- | --- | --- |
|  | | Levene's Test for Equality of Variances | | t-test for Equality of Means | | | | | | |
|  |  | F | Sig. | t | df | Sig. (2-tailed) | Mean Difference | Std. Error Difference | 95% Confidence Interval of the Difference | |
|  |  |  |  |  |  |  |  |  | Lower | Upper |
| LiikkTyopVA_ka.1 | Equal variances assumed | ,125 | ,724 | -,758 | 264 | ,449 | -3,90127 | 5,14499 | -14,03170 | 6,22917 |
|  | Equal variances not assumed |  |  | -,761 | 255,771 | ,447 | -3,90127 | 5,12696 | -13,99770 | 6,19516 |

T-TEST GROUPS=molemmat_hookiet(0 1)

/MISSING=ANALYSIS

/VARIABLES=AskelJaJuoksuTyopVA_ka.1

/CRITERIA=CI(.95).

**T-Test**

| **Group Statistics** | | | | | |
| --- | --- | --- | --- | --- | --- |
|  | molemmat_hookiet | N | Mean | Std. Deviation | Std. Error Mean |
| AskelJaJuoksuTyopVA_ka.1 | Hookie vain alussa 3 pv 10h | 119 | 4594,8964 | 2408,85773 | 220,81963 |
|  | Hookie alussa ja lopussa 3 pv 10h | 147 | 5242,0293 | 3107,58673 | 256,30943 |

| **Independent Samples Test** | | | | | | | | | | |
| --- | --- | --- | --- | --- | --- | --- | --- | --- | --- | --- |
|  | | Levene's Test for Equality of Variances | | t-test for Equality of Means | | | | | | |
|  |  | F | Sig. | t | df | Sig. (2-tailed) | Mean Difference | Std. Error Difference | 95% Confidence Interval of the Difference | |
|  |  |  |  |  |  |  |  |  | Lower | Upper |
| AskelJaJuoksuTyopVA_ka.1 | Equal variances assumed | 4,756 | ,030 | -1,863 | 264 | ,064 | -647,13289 | 347,34546 | -1331,05281 | 36,78702 |
|  | Equal variances not assumed |  |  | -1,913 | 263,532 | ,057 | -647,13289 | 338,31322 | -1313,27384 | 19,00806 |

T-TEST GROUPS=molemmat_hookiet(0 1)

/MISSING=ANALYSIS

/VARIABLES=KevytTyopVA_ka.1

/CRITERIA=CI(.95).

**T-Test**

| **Group Statistics** | | | | | |
| --- | --- | --- | --- | --- | --- |
|  | molemmat_hookiet | N | Mean | Std. Deviation | Std. Error Mean |
| KevytTyopVA_ka.1 | Hookie vain alussa 3 pv 10h | 119 | 97,5642 | 32,59380 | 2,98787 |
|  | Hookie alussa ja lopussa 3 pv 10h | 147 | 96,5430 | 33,05992 | 2,72674 |

| **Independent Samples Test** | | | | | | | | | | |
| --- | --- | --- | --- | --- | --- | --- | --- | --- | --- | --- |
|  | | Levene's Test for Equality of Variances | | t-test for Equality of Means | | | | | | |
|  |  | F | Sig. | t | df | Sig. (2-tailed) | Mean Difference | Std. Error Difference | 95% Confidence Interval of the Difference | |
|  |  |  |  |  |  |  |  |  | Lower | Upper |
| KevytTyopVA_ka.1 | Equal variances assumed | ,039 | ,843 | ,252 | 264 | ,801 | 1,02119 | 4,05113 | -6,95544 | 8,99782 |
|  | Equal variances not assumed |  |  | ,252 | 254,004 | ,801 | 1,02119 | 4,04505 | -6,94493 | 8,98730 |

T-TEST GROUPS=molemmat_hookiet(0 1)

/MISSING=ANALYSIS

/VARIABLES=ReipasTyopVA_ka.1

/CRITERIA=CI(.95).

**T-Test**

| **Group Statistics** | | | | | |
| --- | --- | --- | --- | --- | --- |
|  | molemmat_hookiet | N | Mean | Std. Deviation | Std. Error Mean |
| ReipasTyopVA_ka.1 | Hookie vain alussa 3 pv 10h | 119 | 23,0013 | 15,68981 | 1,43828 |
|  | Hookie alussa ja lopussa 3 pv 10h | 147 | 27,2187 | 18,82878 | 1,55297 |

| **Independent Samples Test** | | | | | | | | | | |
| --- | --- | --- | --- | --- | --- | --- | --- | --- | --- | --- |
|  | | Levene's Test for Equality of Variances | | t-test for Equality of Means | | | | | | |
|  |  | F | Sig. | t | df | Sig. (2-tailed) | Mean Difference | Std. Error Difference | 95% Confidence Interval of the Difference | |
|  |  |  |  |  |  |  |  |  | Lower | Upper |
| ReipasTyopVA_ka.1 | Equal variances assumed | 3,102 | ,079 | -1,955 | 264 | ,052 | -4,21740 | 2,15742 | -8,46535 | ,03054 |
|  | Equal variances not assumed |  |  | -1,992 | 263,768 | ,047 | -4,21740 | 2,11669 | -8,38516 | -,04964 |

T-TEST GROUPS=molemmat_hookiet(0 1)

/MISSING=ANALYSIS

/VARIABLES=IstumTyopVA_ka.1

/CRITERIA=CI(.95).

**T-Test**

| **Group Statistics** | | | | | |
| --- | --- | --- | --- | --- | --- |
|  | molemmat_hookiet | N | Mean | Std. Deviation | Std. Error Mean |
| IstumTyopVA_ka.1 | Hookie vain alussa 3 pv 10h | 119 | 203,7526 | 67,64923 | 6,20140 |
|  | Hookie alussa ja lopussa 3 pv 10h | 147 | 200,8073 | 57,66873 | 4,75644 |

| **Independent Samples Test** | | | | | | | | | | |
| --- | --- | --- | --- | --- | --- | --- | --- | --- | --- | --- |
|  | | Levene's Test for Equality of Variances | | t-test for Equality of Means | | | | | | |
|  |  | F | Sig. | t | df | Sig. (2-tailed) | Mean Difference | Std. Error Difference | 95% Confidence Interval of the Difference | |
|  |  |  |  |  |  |  |  |  | Lower | Upper |
| IstumTyopVA_ka.1 | Equal variances assumed | 4,455 | ,036 | ,383 | 264 | ,702 | 2,94529 | 7,68579 | -12,18797 | 18,07854 |
|  | Equal variances not assumed |  |  | ,377 | 232,609 | ,707 | 2,94529 | 7,81543 | -12,45279 | 18,34337 |

T-TEST GROUPS=molemmat_hookiet(0 1)

/MISSING=ANALYSIS

/VARIABLES=MaIsTyopVA_ka.1

/CRITERIA=CI(.95).

**T-Test**

| **Group Statistics** | | | | | |
| --- | --- | --- | --- | --- | --- |
|  | molemmat_hookiet | N | Mean | Std. Deviation | Std. Error Mean |
| MaIsTyopVA_ka.1 | Hookie vain alussa 3 pv 10h | 119 | 252,5311 | 84,54267 | 7,75001 |
|  | Hookie alussa ja lopussa 3 pv 10h | 147 | 242,3926 | 67,64465 | 5,57924 |

| **Independent Samples Test** | | | | | | | | | | |
| --- | --- | --- | --- | --- | --- | --- | --- | --- | --- | --- |
|  | | Levene's Test for Equality of Variances | | t-test for Equality of Means | | | | | | |
|  |  | F | Sig. | t | df | Sig. (2-tailed) | Mean Difference | Std. Error Difference | 95% Confidence Interval of the Difference | |
|  |  |  |  |  |  |  |  |  | Lower | Upper |
| MaIsTyopVA_ka.1 | Equal variances assumed | 5,053 | ,025 | 1,087 | 264 | ,278 | 10,13852 | 9,33053 | -8,23319 | 28,51024 |
|  | Equal variances not assumed |  |  | 1,062 | 223,488 | ,290 | 10,13852 | 9,54938 | -8,67982 | 28,95687 |
