# Supplementary material for: Moving to business – changes in physical activity and sedentary behavior after multilevel intervention in small and medium-size workplaces
Source: BMC Public Health. 2017 Apr 17;17:319. doi: 10.1186/s12889-017-4229-4 (PMC5392953; doi:10.1186/s12889-017-4229-4)
Supplement: Supplementary file 3 — Statistical analysis to test the difference in change in physical activity and sedentary behavior between the workplaces implementing more or fewer actions than on average and between the workplaces implementing actions at all three levels and just one or two levels. Statistical analysis. (DOCX 105 kb) [file 12889_2017_4229_MOESM3_ESM.docx]

Additional file 3. Statistical analysis to test the difference in change in physical activity and sedentary behavior between the workplaces implementing more or fewer actions than on average and between the workplaces implementing actions at all three levels and just one or two levels

*erojen testaus.

COMPUTE filter_$=(ABS(Total_minutes_ero) <1200 OR MISSING(Total_minutes_ero)).

VARIABLE LABELS filter_$ 'ABS(Total_minutes_ero) <1000 (FILTER)'.

VALUE LABELS filter_$ 0 'Not Selected' 1 'Selected'.

FORMATS filter_$ (f1.0).

FILTER BY filter_$.

EXECUTE.

T-TEST GROUPS=yritys_tp_dik(0 1)

/MISSING=ANALYSIS

/VARIABLES=Total_minutes_ero

/CRITERIA=CI(.95).

**T-Test**

| **Group Statistics** | | | | | |
| --- | --- | --- | --- | --- | --- |
|  | Yrityksen jaoteltuna toimenpiteiden keskiarvon mukaan | N | Mean | Std. Deviation | Std. Error Mean |
| Total_minutes_ero | toimenpiteet alle keskiarvon | 92 | -3,6413 | 189,69192 | 19,77675 |
|  | toimenpiteet yli keskiarvon | 91 | ,0769 | 195,70682 | 20,51565 |

| **Independent Samples Test** | | | | | | | | | | |
| --- | --- | --- | --- | --- | --- | --- | --- | --- | --- | --- |
|  | | Levene's Test for Equality of Variances | | t-test for Equality of Means | | | | | | |
|  |  | F | Sig. | t | df | Sig. (2-tailed) | Mean Difference | Std. Error Difference | 95% Confidence Interval of the Difference | |
|  |  |  |  |  |  |  |  |  | Lower | Upper |
| Total_minutes_ero | Equal variances assumed | ,008 | ,927 | -,131 | 181 | ,896 | -3,71823 | 28,49093 | -59,93531 | 52,49886 |
|  | Equal variances not assumed |  |  | -,130 | 180,678 | ,896 | -3,71823 | 28,49582 | -59,94563 | 52,50917 |

USE ALL.

USE ALL.

COMPUTE filter_$=(ABS(kevyt_ero) <1200 OR MISSING(kevyt_ero)).

VARIABLE LABELS filter_$ 'ABS(Total_minutes_ero) <1000 (FILTER)'.

VALUE LABELS filter_$ 0 'Not Selected' 1 'Selected'.

FORMATS filter_$ (f1.0).

FILTER BY filter_$.

EXECUTE.

T-TEST GROUPS=yritys_tp_dik(0 1)

/MISSING=ANALYSIS

/VARIABLES=kevyt_ero

/CRITERIA=CI(.95).

**T-Test**

| **Group Statistics** | | | | | |
| --- | --- | --- | --- | --- | --- |
|  | Yrityksen jaoteltuna toimenpiteiden keskiarvon mukaan | N | Mean | Std. Deviation | Std. Error Mean |
| kevyt_ero | toimenpiteet alle keskiarvon | 92 | 16,1957 | 182,37802 | 19,01422 |
|  | toimenpiteet yli keskiarvon | 91 | 25,0000 | 160,08505 | 16,78147 |

| **Independent Samples Test** | | | | | | | | | | |
| --- | --- | --- | --- | --- | --- | --- | --- | --- | --- | --- |
|  | | Levene's Test for Equality of Variances | | t-test for Equality of Means | | | | | | |
|  |  | F | Sig. | t | df | Sig. (2-tailed) | Mean Difference | Std. Error Difference | 95% Confidence Interval of the Difference | |
|  |  |  |  |  |  |  |  |  | Lower | Upper |
| kevyt_ero | Equal variances assumed | ,099 | ,754 | -,347 | 181 | ,729 | -8,80435 | 25,37864 | -58,88039 | 41,27170 |
|  | Equal variances not assumed |  |  | -,347 | 178,483 | ,729 | -8,80435 | 25,36057 | -58,84948 | 41,24079 |

USE ALL.

USE ALL.

COMPUTE filter_$=(ABS(reipas_ero) <500 OR MISSING(reipas_ero)).

VARIABLE LABELS filter_$ 'ABS(Total_minutes_ero) <1000 (FILTER)'.

VALUE LABELS filter_$ 0 'Not Selected' 1 'Selected'.

FORMATS filter_$ (f1.0).

FILTER BY filter_$.

EXECUTE.

T-TEST GROUPS=yritys_tp_dik(0 1)

/MISSING=ANALYSIS

/VARIABLES=reipas_ero

/CRITERIA=CI(.95).

**T-Test**

| **Group Statistics** | | | | | |
| --- | --- | --- | --- | --- | --- |
|  | Yrityksen jaoteltuna toimenpiteiden keskiarvon mukaan | N | Mean | Std. Deviation | Std. Error Mean |
| reipas_ero | toimenpiteet alle keskiarvon | 91 | -6,0989 | 101,57592 | 10,64805 |
|  | toimenpiteet yli keskiarvon | 92 | -7,3696 | 109,79969 | 11,44741 |

| **Independent Samples Test** | | | | | | | | | | |
| --- | --- | --- | --- | --- | --- | --- | --- | --- | --- | --- |
|  | | Levene's Test for Equality of Variances | | t-test for Equality of Means | | | | | | |
|  |  | F | Sig. | t | df | Sig. (2-tailed) | Mean Difference | Std. Error Difference | 95% Confidence Interval of the Difference | |
|  |  |  |  |  |  |  |  |  | Lower | Upper |
| reipas_ero | Equal variances assumed | ,003 | ,959 | ,081 | 181 | ,935 | 1,27066 | 15,64075 | -29,59098 | 32,13231 |
|  | Equal variances not assumed |  |  | ,081 | 180,197 | ,935 | 1,27066 | 15,63407 | -29,57874 | 32,12006 |

USE ALL.

T-TEST GROUPS=yritys_tp_dik(0 1)

/MISSING=ANALYSIS

/VARIABLES=rasittava_ero

/CRITERIA=CI(.95).

**T-Test**

| **Group Statistics** | | | | | |
| --- | --- | --- | --- | --- | --- |
|  | Yrityksen jaoteltuna toimenpiteiden keskiarvon mukaan | N | Mean | Std. Deviation | Std. Error Mean |
| rasittava_ero | toimenpiteet alle keskiarvon | 92 | -5,9783 | 79,56005 | 8,29471 |
|  | toimenpiteet yli keskiarvon | 93 | -13,5484 | 70,17672 | 7,27699 |

| **Independent Samples Test** | | | | | | | | | | |
| --- | --- | --- | --- | --- | --- | --- | --- | --- | --- | --- |
|  | | Levene's Test for Equality of Variances | | t-test for Equality of Means | | | | | | |
|  |  | F | Sig. | t | df | Sig. (2-tailed) | Mean Difference | Std. Error Difference | 95% Confidence Interval of the Difference | |
|  |  |  |  |  |  |  |  |  | Lower | Upper |
| rasittava_ero | Equal variances assumed | ,037 | ,848 | ,687 | 183 | ,493 | 7,57013 | 11,02686 | -14,18600 | 29,32625 |
|  | Equal variances not assumed |  |  | ,686 | 179,695 | ,494 | 7,57013 | 11,03434 | -14,20343 | 29,34368 |

USE ALL.

T-TEST GROUPS=yritys_tp_dik(0 1)

/MISSING=ANALYSIS

/VARIABLES=lihasTasap_ero

/CRITERIA=CI(.95).

**T-Test**

| **Group Statistics** | | | | | |
| --- | --- | --- | --- | --- | --- |
|  | Yrityksen jaoteltuna toimenpiteiden keskiarvon mukaan | N | Mean | Std. Deviation | Std. Error Mean |
| lihasTasap_ero | toimenpiteet alle keskiarvon | 92 | 4,8804 | 84,54336 | 8,81425 |
|  | toimenpiteet yli keskiarvon | 92 | -1,0326 | 78,33269 | 8,16675 |

| **Independent Samples Test** | | | | | | | | | | |
| --- | --- | --- | --- | --- | --- | --- | --- | --- | --- | --- |
|  | | Levene's Test for Equality of Variances | | t-test for Equality of Means | | | | | | |
|  |  | F | Sig. | t | df | Sig. (2-tailed) | Mean Difference | Std. Error Difference | 95% Confidence Interval of the Difference | |
|  |  |  |  |  |  |  |  |  | Lower | Upper |
| lihasTasap_ero | Equal variances assumed | ,631 | ,428 | ,492 | 182 | ,623 | 5,91304 | 12,01611 | -17,79575 | 29,62183 |
|  | Equal variances not assumed |  |  | ,492 | 180,951 | ,623 | 5,91304 | 12,01611 | -17,79667 | 29,62275 |

USE ALL.

*toimenpiteiden tasot.

COMPUTE filter_$=(ABS(Total_minutes_ero) <1200 OR MISSING(Total_minutes_ero)).

VARIABLE LABELS filter_$ 'ABS(Total_minutes_ero) <1000 (FILTER)'.

VALUE LABELS filter_$ 0 'Not Selected' 1 'Selected'.

FORMATS filter_$ (f1.0).

FILTER BY filter_$.

EXECUTE.

T-TEST GROUPS=yritys_tptas_dik(0 1)

/MISSING=ANALYSIS

/VARIABLES=Total_minutes_ero

/CRITERIA=CI(.95).

**T-Test**

| **Group Statistics** | | | | | |
| --- | --- | --- | --- | --- | --- |
|  | Yrityksen jaoteltuna toimenpiteiden tasojen mukaan | N | Mean | Std. Deviation | Std. Error Mean |
| Total_minutes_ero | yksipuoliset | 106 | 6,0849 | 183,84906 | 17,85700 |
|  | monipuoliset | 77 | -12,6364 | 203,82881 | 23,22845 |

| **Independent Samples Test** | | | | | | | | | | |
| --- | --- | --- | --- | --- | --- | --- | --- | --- | --- | --- |
|  | | Levene's Test for Equality of Variances | | t-test for Equality of Means | | | | | | |
|  |  | F | Sig. | t | df | Sig. (2-tailed) | Mean Difference | Std. Error Difference | 95% Confidence Interval of the Difference | |
|  |  |  |  |  |  |  |  |  | Lower | Upper |
| Total_minutes_ero | Equal variances assumed | ,512 | ,475 | ,650 | 181 | ,517 | 18,72127 | 28,82291 | -38,15086 | 75,59340 |
|  | Equal variances not assumed |  |  | ,639 | 153,555 | ,524 | 18,72127 | 29,29903 | -39,15995 | 76,60248 |

USE ALL.

USE ALL.

COMPUTE filter_$=(ABS(kevyt_ero) <1200 OR MISSING(kevyt_ero)).

VARIABLE LABELS filter_$ 'ABS(Total_minutes_ero) <1000 (FILTER)'.

VALUE LABELS filter_$ 0 'Not Selected' 1 'Selected'.

FORMATS filter_$ (f1.0).

FILTER BY filter_$.

EXECUTE.

T-TEST GROUPS=yritys_tptas_dik(0 1)

/MISSING=ANALYSIS

/VARIABLES=kevyt_ero

/CRITERIA=CI(.95).

**T-Test**

| **Group Statistics** | | | | | |
| --- | --- | --- | --- | --- | --- |
|  | Yrityksen jaoteltuna toimenpiteiden tasojen mukaan | N | Mean | Std. Deviation | Std. Error Mean |
| kevyt_ero | yksipuoliset | 106 | 14,6698 | 174,40002 | 16,93923 |
|  | monipuoliset | 77 | 28,7013 | 167,58423 | 19,09800 |

| **Independent Samples Test** | | | | | | | | | | |
| --- | --- | --- | --- | --- | --- | --- | --- | --- | --- | --- |
|  | | Levene's Test for Equality of Variances | | t-test for Equality of Means | | | | | | |
|  |  | F | Sig. | t | df | Sig. (2-tailed) | Mean Difference | Std. Error Difference | 95% Confidence Interval of the Difference | |
|  |  |  |  |  |  |  |  |  | Lower | Upper |
| kevyt_ero | Equal variances assumed | ,056 | ,813 | -,546 | 181 | ,586 | -14,03149 | 25,69043 | -64,72275 | 36,65977 |
|  | Equal variances not assumed |  |  | -,550 | 167,556 | ,583 | -14,03149 | 25,52784 | -64,42915 | 36,36617 |

USE ALL.

USE ALL.

COMPUTE filter_$=(ABS(reipas_ero) <500 OR MISSING(reipas_ero)).

VARIABLE LABELS filter_$ 'ABS(Total_minutes_ero) <1000 (FILTER)'.

VALUE LABELS filter_$ 0 'Not Selected' 1 'Selected'.

FORMATS filter_$ (f1.0).

FILTER BY filter_$.

EXECUTE.

T-TEST GROUPS=yritys_tptas_dik(0 1)

/MISSING=ANALYSIS

/VARIABLES=reipas_ero

/CRITERIA=CI(.95).

**T-Test**

| **Group Statistics** | | | | | |
| --- | --- | --- | --- | --- | --- |
|  | Yrityksen jaoteltuna toimenpiteiden tasojen mukaan | N | Mean | Std. Deviation | Std. Error Mean |
| reipas_ero | yksipuoliset | 106 | -,7075 | 100,54717 | 9,76600 |
|  | monipuoliset | 77 | -15,0390 | 112,10163 | 12,77517 |

| **Independent Samples Test** | | | | | | | | | | |
| --- | --- | --- | --- | --- | --- | --- | --- | --- | --- | --- |
|  | | Levene's Test for Equality of Variances | | t-test for Equality of Means | | | | | | |
|  |  | F | Sig. | t | df | Sig. (2-tailed) | Mean Difference | Std. Error Difference | 95% Confidence Interval of the Difference | |
|  |  |  |  |  |  |  |  |  | Lower | Upper |
| reipas_ero | Equal variances assumed | ,737 | ,392 | ,907 | 181 | ,366 | 14,33141 | 15,80511 | -16,85455 | 45,51738 |
|  | Equal variances not assumed |  |  | ,891 | 152,969 | ,374 | 14,33141 | 16,08041 | -17,43695 | 46,09977 |

USE ALL.

T-TEST GROUPS=yritys_tptas_dik(0 1)

/MISSING=ANALYSIS

/VARIABLES=rasittava_ero

/CRITERIA=CI(.95).

**T-Test**

| **Group Statistics** | | | | | |
| --- | --- | --- | --- | --- | --- |
|  | Yrityksen jaoteltuna toimenpiteiden tasojen mukaan | N | Mean | Std. Deviation | Std. Error Mean |
| rasittava_ero | yksipuoliset | 107 | -,5140 | 76,97689 | 7,44164 |
|  | monipuoliset | 78 | -22,5000 | 70,41966 | 7,97346 |

| **Independent Samples Test** | | | | | | | | | | |
| --- | --- | --- | --- | --- | --- | --- | --- | --- | --- | --- |
|  | | Levene's Test for Equality of Variances | | t-test for Equality of Means | | | | | | |
|  |  | F | Sig. | t | df | Sig. (2-tailed) | Mean Difference | Std. Error Difference | 95% Confidence Interval of the Difference | |
|  |  |  |  |  |  |  |  |  | Lower | Upper |
| rasittava_ero | Equal variances assumed | ,309 | ,579 | 1,988 | 183 | ,048 | 21,98598 | 11,06032 | ,16384 | 43,80812 |
|  | Equal variances not assumed |  |  | 2,016 | 173,783 | ,045 | 21,98598 | 10,90660 | ,45953 | 43,51244 |

USE ALL.

T-TEST GROUPS=yritys_tptas_dik(0 1)

/MISSING=ANALYSIS

/VARIABLES=lihasTasap_ero

/CRITERIA=CI(.95).

**T-Test**

| **Group Statistics** | | | | | |
| --- | --- | --- | --- | --- | --- |
|  | Yrityksen jaoteltuna toimenpiteiden tasojen mukaan | N | Mean | Std. Deviation | Std. Error Mean |
| lihasTasap_ero | yksipuoliset | 107 | 6,5794 | 83,09543 | 8,03314 |
|  | monipuoliset | 77 | -4,5455 | 78,88692 | 8,99000 |

| **Independent Samples Test** | | | | | | | | | | |
| --- | --- | --- | --- | --- | --- | --- | --- | --- | --- | --- |
|  | | Levene's Test for Equality of Variances | | t-test for Equality of Means | | | | | | |
|  |  | F | Sig. | t | df | Sig. (2-tailed) | Mean Difference | Std. Error Difference | 95% Confidence Interval of the Difference | |
|  |  |  |  |  |  |  |  |  | Lower | Upper |
| lihasTasap_ero | Equal variances assumed | ,542 | ,463 | ,915 | 182 | ,361 | 11,12489 | 12,15925 | -12,86632 | 35,11611 |
|  | Equal variances not assumed |  |  | ,923 | 168,704 | ,357 | 11,12489 | 12,05618 | -12,67551 | 34,92530 |

USE ALL.
